# Cross-disorder comparative analysis of comorbid conditions reveals novel autism candidate genes

**DOI:** 10.1186/s12864-017-3667-9

**Published:** 2017-04-20

**Authors:** Leticia Diaz-Beltran, Francisco J. Esteban, Maya Varma, Alp Ortuzk, Maude David, Dennis P. Wall

**Affiliations:** 1 Division of Systems Medicine, Department of Pediatrics, School of Medicine, Stanford University, 1265 Welch Road, Stanford, CA 94305-5488 USA; 2 Division of Systems Medicine, Department of Psychiatry, Stanford University, Stanford, CA USA; 3 Systems Biology Unit, Department of Experimental Biology, University of Jaén, Jaén, Spain; 4 Department of Biomedical Data Science, Stanford University, Stanford, CA USA

**Keywords:** Autism Spectrum Disorder, Autism sibling disorders, Gene set enrichment, Process enrichment, Comparative network analysis, Systems biology

## Abstract

**Background:**

Numerous studies have highlighted the elevated degree of comorbidity associated with autism spectrum disorder (ASD). These comorbid conditions may add further impairments to individuals with autism and are substantially more prevalent compared to neurotypical populations. These high rates of comorbidity are not surprising taking into account the overlap of symptoms that ASD shares with other pathologies. From a research perspective, this suggests common molecular mechanisms involved in these conditions. Therefore, identifying crucial genes in the overlap between ASD and these comorbid disorders may help unravel the common biological processes involved and, ultimately, shed some light in the understanding of autism etiology.

**Results:**

In this work, we used a two-fold systems biology approach specially focused on biological processes and gene networks to conduct a comparative analysis of autism with 31 frequently comorbid disorders in order to define a multi-disorder subcomponent of ASD and predict new genes of potential relevance to ASD etiology. We validated our predictions by determining the significance of our candidate genes in high throughput transcriptome expression profiling studies. Using prior knowledge of disease-related biological processes and the interaction networks of the disorders related to autism, we identified a set of 19 genes not previously linked to ASD that were significantly differentially regulated in individuals with autism. In addition, these genes were of potential etiologic relevance to autism, given their enriched roles in neurological processes crucial for optimal brain development and function, learning and memory, cognition and social behavior.

**Conclusions:**

Taken together, our approach represents a novel perspective of autism from the point of view of related comorbid disorders and proposes a model by which prior knowledge of interaction networks may enlighten and focus the genome-wide search for autism candidate genes to better define the genetic heterogeneity of ASD.

**Electronic supplementary material:**

The online version of this article (doi:10.1186/s12864-017-3667-9) contains supplementary material, which is available to authorized users.

## Background

Autism spectrum disorder (ASD) encompasses a group of complex neurodevelopmental disorders characterized, in different ranges, by impaired social interaction, difficulties in verbal and non-verbal communication and restricted, stereotyped and repetitive behaviors. Its symptoms begin in early childhood and persist through adulthood, affecting daily functioning [1].

This lifelong condition, 4 times more common in males than females, is one of the fastest-growing developmental disorders worldwide and its prevalence continues to increase at an alarming rate. In fact, large-scale surveys estimated median rates of increase at 1–2% [1–8]. The US Center for Disease Control and Prevention (CDC) [9] now indicates that 1 in 68 American children have ASD. In addition, the 2014 National Health Interview Survey, conducted by the National Center for Health Statistics (NCHS) estimates that 1 in 45 children ages 3 through 17 have an autism diagnosis [10].

It is clear that ASD is a complex and heterogeneous disorder that arises from the interaction of genetic, neurologic, immunologic and environmental factors [11] with a high and complex heritability, as both rare and common genetic variants contribute to autism risk [12]. The great variation reported in behavioral traits and cognitive profiles make it challenging to define specific genetic risk components [13]. Therefore, despite recent scientific advances shedding light into the molecular agents and biological mechanisms responsible for ASD, contributing to the discovery and validation of its causative genes [14], the exact factors still remain elusive and no unifying hypothesis about the molecular pathology of autism has emerged.

Interestingly, several large-scale clinical studies have confirmed the high rate of comorbidity associated with ASD. These comorbid conditions represent an additional burden of illness [15]. Indeed, approximately more than 70% of individuals diagnosed with autism have concurrent medical conditions with significantly higher frequency than in neurotypical populations [12]. Some of these disorders, like epilepsy or depression, can first appear in puberty or even later in life, compounding lifelong impairment. Almost 45% of individuals with autism are also affected by intellectual disability, 28–44% have been diagnosed with attention deficit hyperactivity disorder, 12–70% have clinical depression, 8–30% of ASD individuals have epilepsy, 42–56% have anxiety and 9–70% of manifest gastrointestinal problems [12].

These high rates of comorbidity are not surprising considering the overlap of ASD symptoms with many other human disorders, either neurological in nature or not. This suggests a testable hypothesis: disorders with an elevated level of co-occurrence with autism may have many genes in common with ASD and therefore an overlap in the biological processes involved. Thus, the detection of key genes present in the intersection between ASD and several concurrent disorders (behaviorally related, comorbid or both) may help decipher common molecular mechanisms and/or a shared pathophysiology and, ultimately, yield powerful insights in the understanding of autism etiology.

In the present work, we performed a comprehensive cross-disorder analysis comparing autism with 31 comorbid conditions with the aim of quantifying their overlap at the level of molecular physiology, specifically focusing on biological processes and gene networks. We used a systems biology approach to robustly characterize disease genes, identify the comorbid disorders most closely related with autism and quantify and explore the intersection. By implementing a two pronged strategy that leverages both gene function and network connectivity [16] we took advantage of the prior knowledge from related conditions to predict new genes of possible relevance to ASD etiology. Finally, we utilized transcriptome expression profiling experiments to validate our predictions, by identifying significant differential expression of our novel candidate genes in these high-throughput studies.

## Methods

### Diseases and gene lists

To obtain a robust set of related conditions, we leveraged the results of research studies that investigated autism comorbidity occurring at a significantly higher frequency in ASD patients than in an age-matched control population, using a population- derived sample [17–19], electronic records [19–23] and review papers [19, 24]. We extracted all the ICD-9 codes of autism and its comorbid conditions in these studies and, when ICD-9 code lists were not directly available, we matched the co-occurring conditions mined from these sources to their corresponding codes and references under the ICD-9 system, broadly used in healthcare [25]; then, we mapped each ICD-9 code in our comorbid disorder list to MeSH (Medical Subject Headings form from U.S. National Library of Medicine) terms in order to facilitate the subsequent automated gene search. For instance, the MeSH Term “Anxiety disorders” was matched to the ICD-9 code 300.02 consistent with the ICD-9 reference “Generalized anxiety disorder”, while the MeSH Term “Depressive disorder” corresponded to the 296.3 ICD-9 code with expanded description of “major depressive disorder recurrent episode”.

Next, we generated lists of disorder-related genes by using two powerful text mining tools, Phenopedia and Genehawk, which text mine disease-to-gene relationships in the bibliome. Phenopedia [26], is a web-based application that gathers human genetic associations from literature through a database constantly updated from Pubmed, using either genes or diseases as the starting point. The complete method is described in Yu et al. [26]. Genehawk [27] is a gene-disorder-publication database that collects and ranks associations between genes and diseases, built on evidences from all publication abstracts available via PubMed, as well as the type of study itself. For a given disorder, Genehawk retrieves all related abstracts, filters out those with specific genetic test results and mines gene symbols and maps these to unique identifiers; finally, the obtained results are ranked to assess their significance taking into account the number of supporting evidences, the article structure (review or hypothesis) and the strength of the publication (journal impact factor and year of publication). A complete explanation of this method can be found in Jung et al. [27]. Since both sources, Phenopedia and Genehawk, employ MeSH terms for their automatic exploration of Pubmed, we matched our comorbid disorder ICD-9 code list to this controlled vocabulary thesaurus used for article indexing, as pointed out previously. For ASD, we completed our resulting list of associated genes by adding the autism genes included in SFARI gene [28], as well as those reported as candidates in Iossifov et al. [21] and De Rubeis et al. [29].

### Disease-gene cluster and bootstrap validation

We then converted the obtained seed list into a matrix of binary gene presence/absence with respect to each disorder. The matrix was analyzed using the Jaccard coefficient in MATLAB® to build a gene-based dendrogram of all comorbid disorders. The Jaccard statistic, defined by the size of the intersection divided by the size of the union of sample sets, was originally conceived for pattern discovery with binary matrices and computes the similarity and diversity among sample sets without considering the shared absence of a characteristic as evidence for relatedness.

For assessing clusterwise stability and validity of the groupings within the disease relationship tree we used clusterboot(), an integrated function of the ‘fpc’ package in R [30]. We resampled with replacement from the original data by using a non-parametric bootstrapping method (B = 1000 runs) with the aim of generating bootstrap matrices and clusters and iteratively utilized the Jaccard coefficient to measure the structural similarity of the resampled trees with the tree derived from the original data. We considered the mean of the Jaccard coefficients, calculated per permutation as the overall similarity between the original and iterated data, as the index of cluster’s stability and validity. In order to match the total number of clusters obtained in the observed disease relationship tree, we set for each permutation the number of subsets, k, to 6. Then, those clusters supported by a Jaccard coefficient greater than 0.6 were considered robust and stable, while values approaching 1.0 exhibited the highest stability. Jaccard coefficient values equal or lower than 0.5 were considered not stable and, thus, not taken into account for the analysis. A complete explanation of this method can be found in this study by Hennig [31]. These cluster stability analyses were complemented with a classical multidimensional scaling approach that projects our dissimilarity data onto its first two principal dimensions, generated by the ‘showplots’ argument of Clusterboot() function.

### Generation of molecular networks

We used STRING (Search Tool for the Retrieval of Interacting Genes/Proteins) version 10 [32] to generate networks for the gene lists of the concurrent conditions most closely related with ASD. The networks were created using the default settings in STRING and the lists of edges were derived from all the available lines of evidence: Neighborhood, Gene Fusion, Co-occurrence, Co-expression, Experiments, Databases and Textmining. It is worth highlighting that, in STRING, every source of interaction evidence is benchmarked and calibrated against prior knowledge, according to the manually curated information provided by the Kyoto Encyclopedia of Genes and Genomes (KEGG) pathway maps [33]. The complete method has been described by Szklarczyk et al. [32]. The returned gene interactions were used for subsequent analysis in our network-driven search for autism candidate genes.

### Biological process enrichment

To identify the biological processes for which the comorbid disorders most closely related with autism were enriched, we utilized DAVID Bioinformatics Resources (Database for Annotation, Visualization and Integrated Discovery) version 6.7 [34], a high-throughput data-mining environment. This web-accessible functional annotation tool for gene ontology (GO) enrichment analysis embodies an integrated biological knowledge database and analytical implements to automatically extract biological features/meanings associated with large lists of genes. Further information regarding DAVID protocol is detailed in [34]. For our analysis, we employed the “Functional Annotation” tool that basically provides batch annotation and gene-GO term enrichment analysis to emphasize the most important GO terms related with a specified gene list. By choosing the GO fat categories,“GOTERM-BP-FAT” option, to report the enrichment results, we are selecting a subset of the more general GO term; hence, the broadest terms are filtered so that they will not overshadow the more specific ones. In order to evade over counting duplicated genes, DAVID performs Fisher Exact statistics on corresponding DAVID gene IDs by which all redundancies in original IDs are eliminated. All the results displayed in the Functional Chart Report did pass the established thresholds (by default, Max. Prob. < =0.1 and Min. Count > =2) so as to ensure only the statistically significant outcomes are showed. Finally, only those biological processes with a false discovery rate (FDR) score below 0.05 were selected as strongly enriched, according to their statistical significance after multiple test correction.

### Expression analysis

From Gene Expression Omnibus (GEO) [35] we downloaded data from three independent experiments, GSE18123 (gpl570) [36], GSE25507 [37] and GSE42133 [38], in order to validate our autism candidate genes. Additional file 1 (Table S1) summarizes the information about the datasets selected. Raw data of Affymetrix datasets, GSE18123 (gpl570) and GSE25507, was preprocessed and RMA normalized using ‘affy’ package in R [39] and Bioconductor [40], while with the Illumina dataset GSE42133 we employed the preprocessed data provided in GEO database, Log2 transformed and quantile normalized using Illumina GenomeStudio® software (version 1.1.1) and ‘Lumi’ package in R and Bioconductor [41]. Additional file 2 (Figure S1) shows the distribution of the samples after preprocessing, median-centered values indicate that the data are normalized and cross-comparable. All expression analyses were done using mt.teststat function from “multtest” package in R and Bioconductor [42]; to increase the test power for samples with unequal sample size and variance, we performed a *t*-test based on two-sample Welch t-statistics to determine the difference in signal between the ASD and control group. Finally, we performed multiple test correction to the unadjusted *p*-values from the comparative analyses by calculating *q*-values, a measure of significance in terms of the FDR [43].

### Functional analysis of ASD candidate genes

Our candidate genes identified to be differentially expressed in all three experiments were uploaded into the QIAGEN® Ingenuity® Pathway Analysis (IPA®) software, in order to explore gene connectivity and related biological functions both within and across disease. IPA® uses a human-curated pathways knowledge base containing genes, proteins and RNAs to retrieve biological interactions and associate biological functions and disorders with experimental results, providing statistical support for gene-to-gene associations. We generated networks for our ASD candidate genes using an edge rank score (p-score = −log10 (*p*-value)) that designated the likelihood of the concurrent or interacting genes by random chance. A rank score value greater than 3 (*p* < 0.001) denoted an edge linking two genes as a statistically relevant not random association, with more than 99.9% confidence. Additionally, we performed a “disease and function” analysis to test whether our ASD gene candidates were enriched in specific human disorders and investigated their role in the context of statistically significant biological processes, pathways and networks. IPA® performs a Fisher exact test to calculate *p*-values that define the significance of the association between a focus gene and a biological process or pathway; thus, those biological processes with *p*-values ≤0.05 are considered as statistically significantly enriched with genes of interest.

## Results

### The multi-disorder component of ASD

**Fig. 1:**
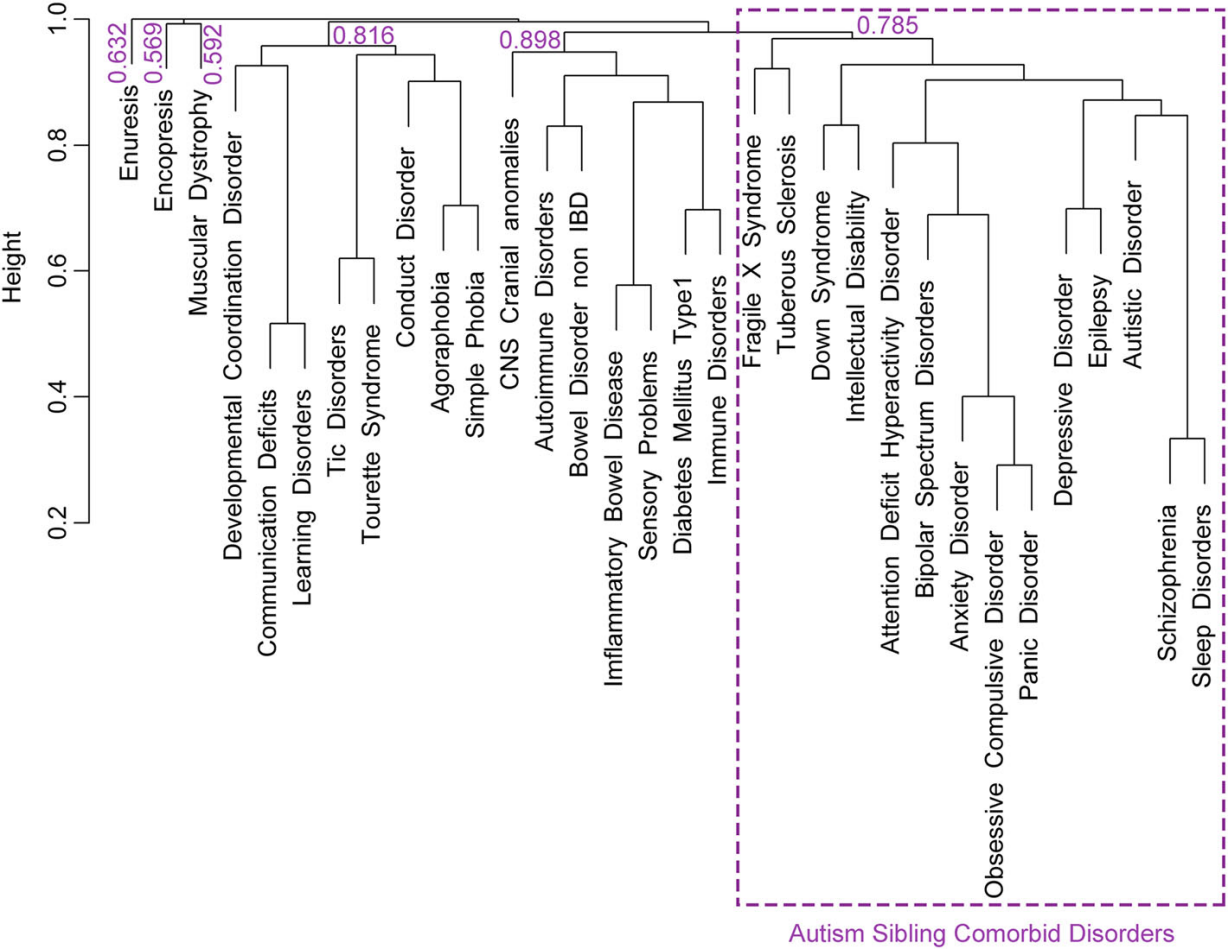
Gene-based phylogeny of autism and related co-ocurring conditions, generated using the Jaccard Coefficient. The group containing autism is highlighted and referred to in the text as “Autism sibling comorbid disorders”. Bootstrap stability indices are also provided for each subgroup

We retrieved from the literature [17–24] 132 medical conditions concurrent with autism that were matched with their corresponding codes and references under ICD-9 system [25] and later consolidated into a defined set of 31 disorders comorbid with ASD (See Additional file 3: Table S2). Using Phenopedia [26] and Genehawk [27], we generated lists of genes associated to each comorbid condition and, as pointed out previously, in the case of autism, we completed its gene list by adding the ASD candidate genes included in SFARI gene [28], Iossifov et al. [21] and De Rubeis et al. [29]. The total number of genes utilized for this study are detailed in Additional file 4: Table S3. By converting the retrieved gene lists into a binary matrix of gene presence/absence, we were able to generate a disorder phylogeny using the Jaccard Coefficient (Fig. 1). The tree obtained grouped autism with 13 disorders that we called “sibling” comorbid disorders of ASD, including epilepsy, intellectual disability, fragile X syndrome, schizophrenia, depressive disorder, bipolar disorder and attention deficit hyperactivity disorder (ADHD), among others. Cluster wise validity and stability within the tree was assessed by means of a non-parametric bootstrap procedure (1000 runs) that yielded a mean Jaccard value of 0.785 for our autism sibling comorbid disorders cluster (See Additional file 5: Table S4, also Additional file 6: Figure S2). Thus, we considered this sibling group as stable and statistically robust and focused on this group for subsequent analyses.

With the aim of exploring genetic overlap between autism and its sibling comorbid conditions, we used the tool STRING [32] to generate gene networks for each member of the ASD sibling group (edge summary included in Additional file 7: Table S5). Of the 1066 genes present in our seed list for ASD, 710 have also been linked to at least one other autism sibling disorder (the multi disorder autism gene set, and the sibling comorbid conditions where they are found, are detailed in Additional file 8: Table S6). This multi disorder autism gene set (MDAG) conforms a highly interconnected subcomponent of the ASD gene network (Additional file 9: Figure S3), suggesting common molecular mechanisms and shared biological functions among the MDAG members. To test this, we utilized DAVID [34] to identify significant enrichment of MDAG genes in biological processes (BP). A total of 378 BP had significant overrepresentation following FDR multiple test correction (top 30 BP are listed in the Table 1), significant if FDR < 0.05; the complete list of BP for which the MDAG genes are enriched can be found in Additional file 10: Table S7.

**Table 1:** Top 30 biological processes for which the multi-disorder component of the autism gene set (MDAG) were enriched

Biological process	# MDAG genes	*p*-Value	FDR
Transmission of nerve impulse	119	1.18E-71	2.18E-68
Synaptic transmission	110	2.53E-70	4.67E-67
Behavior	123	1.07E-59	1.97E-56
Cell-cell signaling	137	7.08E-59	1.31E-55
Regulation of system process	84	2.00E-41	3.70E-38
Neurological system process	164	7.03E-39	1.30E-35
Regulation of neurological system process	57	4.56E-36	8.42E-33
Learning or memory	49	4.57E-35	8.43E-32
Regulation of transmission of nerve impulse	54	8.64E-34	1.59E-30
Regulation of synaptic transmission	51	2.12E-32	3.91E-29
Neuron differentiation	85	2.91E-30	5.38E-27
Neuron development	74	1.13E-29	2.08E-26
Neuron projection development	61	1.40E-26	2.59E-23
Second-messenger-mediated signaling	58	4.18E-26	7.71E-23
Cyclic-nucleotide-mediated signaling	44	7.72E-26	1.43E-22
Cell morphogenesis involved in neuron differentiation	54	2.48E-25	4.57E-22
Cell morphogenesis involved in differentiation	58	3.25E-25	5.99E-22
G-protein signaling, coupled to cyclic nucleotide second messenger	41	4.61E-25	8.51E-22
Learning	31	1.12E-24	2.07E-21
Neuron projection morphogenesis	53	4.86E-24	8.98E-21
Response to endogenous stimulus	73	6.40E-24	1.18E-20
Regulation of secretion	51	1.92E-23	3.54E-20
Axonogenesis	49	1.21E-22	2.23E-19
Feeding behavior	31	2.00E-22	3.69E-19
Response to organic substance	97	5.25E-22	9.70E-19
Intracellular signaling cascade	135	1.40E-21	2.59E-18
Cell projection organization	66	1.65E-21	3.04E-18
Cell projection morphogenesis	53	4.54E-21	8.39E-18
Regulation of cellular localization	53	8.10E-21	1.50E-17
Cell motion	74	2.12E-20	3.92E-17

### Biological process-driven search for novel ASD candidates

The large extent of genetic overlap between autism and several of its sibling conditions may rely on specific dysregulations of any or all the biological processes for which the MDAG is enriched. Therefore, other genes associated to any of the 378 statistically significant processes that have not yet been linked to ASD could be regarded as possible novel candidates for autism. To address this premise, the gene lists of all the ASD sibling disorders were mined to identify and retrieve a non-redundant set of 1588 process-based candidates (PBC); 34 processes were not found among the genes in the autism sibling disorders (See Additional file 10: Table S7). All other enriched processes returned 2 or more predictions all of which are implicated in at least 2 autism sibling disorders, but not found in our original gene candidate list for ASD. The complete list of 1588 process-based candidates can be found in Additional file 11: Table S8.

To empirically test the importance of our process-based candidates, we checked whether they were significantly differentially regulated in autistic patients versus healthy controls, using the three independent GEO experiments described above (GSE18123gpl570, GSE25507 and GSE42133) [36–38]. Since our foremost interest was to confirm our PBC, we performed multiple test correction to the unadjusted *p*-values obtained from the analyses by calculating *q*-values (see Additional file 12: Figure S4), an FDR-based measure of significance. For each experiment, we considered the number of PBC present in the array as the total number of hypotheses, as previously done in [16]. The resultant number of differentially expressed PBC with *q*-value <0.05 are summarized in Table 2, where there are 1058 significant PBC for GSE18123gpl570, 626 for GSE25507 and 269 in the case of GSE42133; a total of number of 80 significant PBC constituted the overlap among the three datasets. The identities of the differentially expressed PBC in each experiment, along with their corresponding *q*-values, the biological processes where they are involved and the comorbid disorders where they are implicated, can be also found in Additional file 11: Table S8.

**Table 2:** Number of significantly differentially expressed process and network based candidates in the datasets

	GSE18123gpl570	GSE25507	GSE42133	All datasets
# significant PBC (q < 0.05)	1058	626	269	80
# significant NBC (q < 0.05)	1210	691	298	91
# significant PBC∩NBC (q < 0.05)	925	532	214	64
# significant PBC∩NBC in 3 or more siblings (q < 0.05)	330	183	69	19

### Network-driven search for new autism genes

Using data derived from STRING [32], we constructed gene networks for each of the autism sibling disorders with the purpose of exploring the surrounding members of the MDAG genes, specifically focusing on their first neighbors that were not included in our original ASD candidate list. This analysis yielded 1794 network-based candidates (NBC), directly linked to a member of the MDAG but not known yet as relevant for autistic disorder. From the total set of genes constituting the NBC, 233 candidates occur in at least 5 sibling conditions, 74 are present in 7 or more siblings, 29 in 8 or more, 13 in 9 autism siblings, 3 in 10 autism sibling disorders (MAGI2, NR3C1, SLC1A2) and one (SLC1A2) present in 12 siblings. The complete list of 1794 network-based candidates can be found in Additional file 13: Table S9.

We leveraged the same mRNA expression datasets as before to calculate *q*-values and verify whether our network-based candidates exhibited significantly different gene expression in individuals with autism when compared to normal controls (see Additional file 12: Figure S4). We validated the NBC by testing for significant differential expression in each of the three separate microarray experiments, GSE18123gpl570, GSE25507 and GSE42133. We treated each test of the NBC as a separate experiment and adjusted for multiple testing each time by computing the q-value for the total number of NBC genes found on the separate arrays, 1210, 691, 298, respectively. Table 2 shows the number of NBC found to be significantly differentially regulated (*q*-value <0.05) in each experiment. A total of 91 significant NBC were found in common among the three gene expression datasets. Their identities, *q*-values, MDAG interactors and comorbid disorders in which they play a role, can be also found in Additional file 13: Table S9.

### Intersection of PBC and NBC to prioritize autism candidate genes

We intersected our two computational strategies to triangulate on the set of genes that were independently predicted and verified by both approaches. A total of 1358 genes formed the overlap of PBC and NBC (PBC∩NBC); the total number of significant differentially expressed candidates, with *q*-value < 0.05, predicted in each experiment by both approaches is 925, 532 and 214 genes for GSE18123gpl570, GSE25507 and GSE42133 respectively (Table 2), with a total of 64 significant candidates overlapping across all three experiments. The identities of these candidate genes are detailed in Additional file 14: Table S10, along with the biological processes where they participate, their MDAG interactors and the comorbid disorders associated with them. Next, we cut down the size of the overlap by removing those genes that occur in 2 or fewer autism sibling disorders. This is based on the premise that genes with numerous independent associations to our sibling comorbid disorders are more likely to participate in typical neurodevelopmental processes and functions. From the 1358 genes present in the overlap (PBC∩NBC), only 489 candidates predicted by both strategies occurred in 3 or more siblings. Table 2 also shows, for each dataset, the number of differentially expressed candidates independently predicted and verified by both strategies occurring in 3 or more autism sibling disorders: 330 for GSE18123gpl570, 183 for GSE25507 and 69 in the case of GSE42133.

**Fig. 2:**
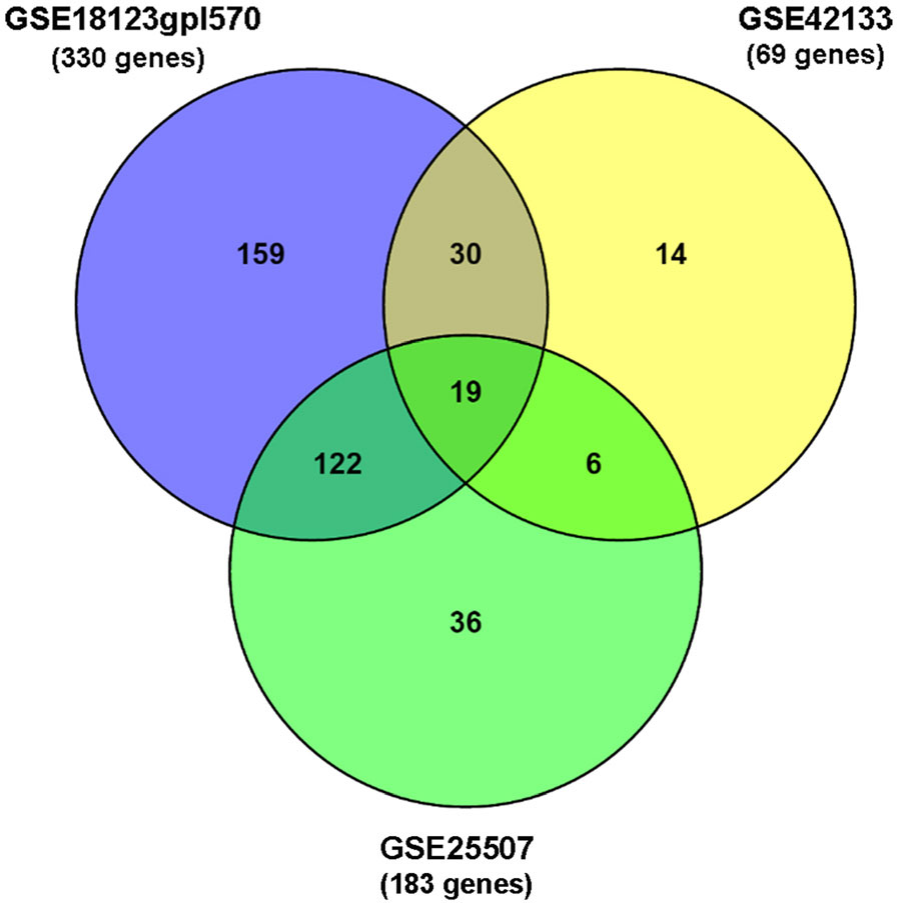
Venn diagram showing the overlap in the number of significant differentially regulated process and network based candidate genes (PBC∩NBC) occurring in 3 or more autism sibling comorbid disorders for the three datasets. Only 19 candidate genes are present in all three transcriptome experiments

Finally, with the aim of obtaining a definitive set of candidates, we intersected the differentially expressed genes obtained from the analysis of the three GEO datasets occurring in 3 or more sibling comorbid disorders; this yielded an overlap of 19 genes (Table 3). The overlap across the three sets of differentially expressed genes is shown in Fig. 2.

**Table 3:** List of the 19 candidate genes significantly differentially expressed in the three experiments and the disorders where they are implicated

Genes	Sibling disorders	# Disorders
ADAM10	Bipolar Spectrum Disorders, Down Syndrome, Sleep Disorders	3
ADCY9	Bipolar Spectrum Disorders, Depressive Disorder, Epilepsy, Schizophrenia, Sleep Disorders	5
ADCYAP1R1	Anxiety Disorder, Bipolar Spectrum Disorders, Obsessive Compulsive Disorder, Panic Disorder	4
AKT1	Bipolar Spectrum Disorders, Depressive Disorder, Epilepsy, Fragile X Syndrome, Schizophrenia, Tuberous Sclerosis	6
ATN1	Epilepsy, Fragile X Syndrome, Intellectual Disability, Schizophrenia, Sleep Disorders	5
DGCR8	Depressive Disorder, Fragile X Syndrome, Schizophrenia, Sleep Disorders	4
DLGAP4	Anxiety Disorder, Bipolar Spectrum Disorders, Obsessive Compulsive Disorder, Panic Disorder, Schizophrenia, Sleep Disorders	6
HSPA1L	Bipolar Spectrum Disorders, Depressive Disorder, Schizophrenia	3
KCNH2	Epilepsy, Intellectual Disability, Schizophrenia, Sleep Disorders	4
MEGF10	Bipolar Spectrum Disorders, Schizophrenia, Sleep Disorders	3
MMP2	Epilepsy, Sleep Disorders, Tuberous Sclerosis	3
NDE1	Bipolar Spectrum Disorders, Epilepsy, Intellectual Disability, Schizophrenia	4
NPPB	Anxiety Disorder, Bipolar Spectrum Disorders, Obsessive Compulsive Disorder, Panic Disorder, Sleep Disorders	5
NRP1	Anxiety Disorder, Bipolar Spectrum Disorders, Obsessive Compulsive Disorder, Panic Disorder, Sleep Disorders	5
PPP3CB	Attention Deficit Hyperactivity Disorder, Schizophrenia, Sleep Disorders	3
PRKG1	Attention Deficit Hyperactivity Disorder, Fragile X Syndrome, Schizophrenia, Sleep Disorders	4
SLC29A2	Depressive Disorder, Epilepsy, Sleep Disorders	3
SMARCA2	Epilepsy, Intellectual Disability, Schizophrenia, Sleep Disorders	4
VIPR2	Anxiety Disorder, Bipolar Spectrum Disorders, Depressive Disorder, Down Syndrome, Epilepsy, Intellectual Disability, Obsessive Compulsive Disorder, Panic Disorder, Schizophrenia	9

Additionally, this final set of 19 candidates was loaded into IPA® to explore connectivity and biological function. The analysis performed associated our 19 candidate genes to four Top Canonical Pathways (Table 4), related to brain development and function, neurodegeneration and behavior and known to be relevant in the molecular pathology of ASD. We also conducted a “Diseases & Function” analysis for the 19 genes that linked them with several significant annotations; Table 5 shows the most compelling examples and the candidate genes involved in each biological function.

**Table 4:** Top canonical pathways for which the 19 candidates genes are enriched, according to Ingenuity® Pathway Analysis (IPA®)

Canonical pathway	*p*-value	Overlap
eNOS signaling	9.45–06	3.0% 4/135
Gap junction signaling	1.47–05	2.6% 4/151
Axonal guidance signaling	5.55–05	1.2% 5/427
Glucocorticoid receptor signaling	1.46–04	1.5% 4/272

**Table 5:** Significant functional annotations of our final set of candidate genes according to Ingenuity® Pathway Analysis (IPA®)

Diseases or functions annotation	*p*-value	Candidate genes involved
Proliferation of nervous tissue cell lines	2.07E-05	AKT1,NRP1
Action potential of embryonic stem cell lines	1.02E-03	KCNH2
Arrest in growth of nervous tissue cell lines	1.02E-03	NRP1
Formation of cranium	1.02E-03	MMP2
Quiescence of nervous tissue cell lines	1.02E-03	NRP1
Schizophrenia	1.55E-03	AKT1,KCNH2,PPP3CB,SMARCA2
Generation of plasmacytoid dendritic cells	4.08E-03	AKT1
Induction of CD4+ T-lymphocytes	5.10E-03	PRKG1
Induction of Th17 cells	5.10E-03	PRKG1
Binding of cells	5.40E-03	MMP2,NPPB,NRP1
Permeability of blood–brain barrier	6.11E-03	MMP2
Gene silencing	8.14E-03	SMARCA2
Loss of neurons	1.32E-02	ATN1
Rasmussen’s encephalitis	1.42E-02	PPP3CB
Cognition	1.52E-02	AKT1
Neuropathic pain	2.12E-02	KCNH2
Apoptosis of dendritic cells	2.22E-02	AKT1
Release of nitric oxide	2.32E-02	AKT1
Quantity of neurons	2.42E-02	ATN1
Transcription of RNA	2.84E-02	AKT1,ATN1,SMARCA2
Epilepsy	3.13E-02	HSPA1L,NPPB

**Fig. 3:**
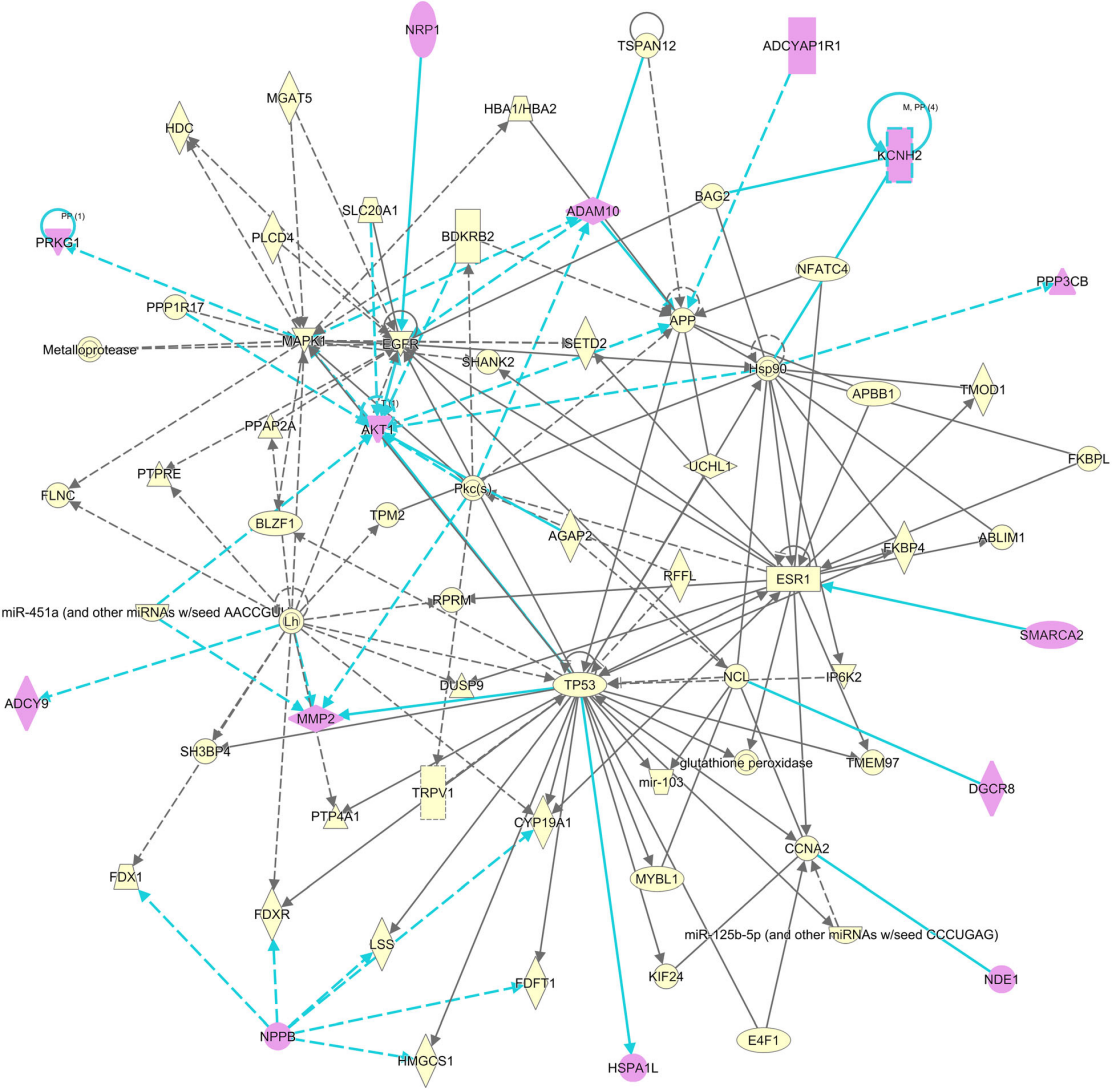
Statistically significant biological network obtained through Ingenuity Pathway Analysis (IPA®). 14 of our 19 candidate genes are tightly connected and interact in neurological processes and conditions detailed in Table 6. Our candidate genes are highlighted in purple and their interactions in turquoise

Regarding gene connectivity, IPA® provided a statistically robust network (score = 31), shown in Fig. 3, where 14 genes from the original 19 candidates are interacting with other molecules in several significant neurological processes involved in normal brain growth and development, such as proliferation of neuronal cells, formation and branching of neurites, migration of neurons, among others (see Table 6). Dysregulation of any of these candidates may affect crucial brain processes since many of them interact with genes already included in our original seed list for autism (APP, CYP19A1, ESR1, MAPK1, SETD2, SHANK2, TRPV1), some of them being highly interconnected nodes within the network (ESR1, APP, MAPK1). In addition, important neurological processes such as cognition, learning and memory may be altered since several of our candidates are linked to key network genes (ESR1, Pkcs, AKT1), implicated in postsynaptic density and glutamatergic synapses, and, hence, in synaptic plasticity. Furthermore, our candidate genes also seem to be involved in pathways where central genes within the network, such as MAPK1, ESR1, Pkcs, TP53, APP and EGFR, are thought to regulate molecular functions associated with multiple aspects of social and anxiety-related behaviors, mood outcomes and impaired long-term memory, cognitive degeneration and neurological dysfunction. This network also showed an interesting connection between genes linked to ASD and other neurological conditions and endocrine hormones of the hypothalamic-pituitary-gonadal axis, such as the luteinizing hormone (Lh). The neurological functions statistically significantly enriched in the network are described in Table 6, along with the genes implicated in each process and their corresponding *p*-values.

**Table 6:** Significant diseases and functions enriched in the biological network (Fig. 3) obtained through Ingenuity® Pathway Analysis (IPA®)

Diseases or functions annotation	*p*-Value	Molecules
Proliferation of neuronal cells	1.95E-06	**ADAM10**, **ADCYAP1R1**, **AKT1**, APBB1, APP, CYP19A1, EGFR, ESR1, FKBP4, HBA1/HBA2, MAPK1, MMP2, **NDE1**, NFATC4, **NRP1**, Pkc(s), TP53
Growth of neurites	3.24E-06	**ADAM10**, **AKT1**, APBB1, APP, CYP19A1, EGFR, ESR1, FKBP4, HBA1/HBA2, MAPK1, MMP2, NFATC4, **NRP1**, pkc(s), TP53
Interphase of brain cells	8.22E-06	**ADCYAP1R1**, APP, TP53
Outgrowth of neurites	1.78E-05	**ADAM10**, **AKT1**, APBB1, APP, EGFR, ESR1, FKBP4, HBA1/HBA2, MAPK1, NFATC4, **NRP1**, Pkc(s), TP53
Behavior	1.98E-05	**ADAM10**, AGAP2, APBB1, APP, CYP19A1, ESR1, HBA1/HBA2, HDC, MAPK1, mir-103, MYBL1, NFATC4, **NPPB**, Pkc(s), **PPP3CB**, **PRKG1**, SHANK2, TP53, TRPV1, UCHL1
Alzheimer’s disease	5.88E-05	**ADAM10**, APBB1, APP, ESR1, FDFT1, HBA1/HBA2, LSS, mir-103, miR-125b-5p (and other miRNAs w/seed CCUGAG), **MMP2**, NFATC4, Pkc(s), TP53, UCHL1
Microtubule dynamics	7.64E-05	ABLIM1, **ADAM10**, **AGAP2**, **AKT1**, APP, CYP19A1, DUSP9, EGFR, ESR1, FKBP4, Hsp90, KIF24, MAPK1, **NDE1**, NFATC4, **NRP1**, Pkc(s), **PPP3CB**, **PRKG1**, PTPRE, TP53, UCHL1
Organization of cytoskeleton	7.78E-05	ABLIM1, **ADAM10**, AGAP2, **AKT1**, APP, CYP19A1, DUSP9, EGFR, ESR1, FKBP4, FLNC, Hsp90, KIF24, MAPK1, MGAT5, **NDE1**, NFATC4, **NRP1**, Pkc(s), **PPP3CB**, **PRKG1**, PTPRE, TP53, UCHL1
Branching of neurites	7.87E-05	**ADAM10**, AGAP2, **AKT1**, APP, CYP19A1, NFATC4, **NRP1**, **PRKG1**, TP53
Entry into S phase of cerebral cortex cells	1.18E-04	**ADCYAP1R1**, APP
Anxiety	1.65E-04	**ADCYAP1R1**, APP, HDC, MAPK1, NFATC4, SHANK2, TRPV1
Branching of cells	1.86E-04	**ADAM10**, AGAP2, **AKT1**, APP, BDKRB2, CYP19A1, NFATC4, **NRP1**, Pkc(s), **PRKG1**, TP53
Hyperactive behavior	1.92E-04	**ADCYAP1R1**, **AKT1**, APP, ESR1, **PPP3CB**, SHANK2
Development of central nervous system	2.07E-04	**ADAM10**, **ADCYAP1R1**, **AKT1**, APBB1, APP, CYP19A1, EGFR, MAPK1, **NDE1**, **PRKG1**, SETD2, TP53, TRPV1
Interphase of neural precursor cells	2.67E-04	**ADCYAP1R1**, TP53
Conditioning	2.87E-04	**ADCYAP1R1**, APP, ESR1, MAPK1, **MMP2**, TRPV1, UCHL1
Firing of neurons	2.97E-04	APP, MAPK1, **NPPB**, TRPV1
Locomotion	3.09E-04	AGAP2, APP, CYP19A1, ESR1, NFATC4, **PPP3CB**, TMOD1, TP53, UCHL1
Formation of brain	3.37E-04	**ADAM10**, **ADCYAP1R1**, APBB1, APP, CYP19A1, EGFR, **NDE1**, **PRKG1**, SETD2, TP53, TRPV1
Cell viability of neuroglia	3.48E-04	**AKT1**, APP, EGFR, TP53
Cell death of sympathetic neuron	3.96E-04	AGAP2, **AKT1**, APP, Pkc(s), TP53
Morphogenesis of neurites	4.00E-04	**ADAM10**, AGAP2, **AKT1**, APP, CYP19A1, EGFR, NFATC4, **NRP1**, **PRKG1**, TP53
Neuritogenesis	4.44E-04	**ADAM10**, AGAP2, **AKT1**, APP, CYP19A1, EGFR, NFATC4, **NRP1**, **PRKG1**, PTPRE, TP53, UCHL1
Formation of forebrain	5.97E-04	**ADAM10**, **ADCYAP1R1**, APBB1, APP, **NDE1**, **PRKG1**, SETD2
Migration of neurons	9.72E-04	**ADAM10**, APBB1, DGCR8, EGFR, **NDE1**, **NRP1**, **PRKG1**
Emotional behavior	1.06E-03	APP, CYP19A1, ESR1, MAPK1, **NPPB**, SHANK2, TRPV1
Schizophrenia spectrum disorder	1.21E-03	**AKT1**, APP, EGFR, ESR1, **KCNH2**, mir-103, Pkc(s), **PPP3CB**, SHANK2, **SMARCA2**
Long-term potentiation	1.22E-03	**ADCYAP1R1**, APP, CYP19A1, EGFR, MAPK1, Pkc(s), SHANK2, TRPV1
Cognition	1.52E-02	**AKT1**

Our candidate genes are highlighted in bold

Using this novel two-pronged computational approach, we were able to discover a final set of 19 ASD candidate genes that have been predicted by both strategies (network and process-based) that occur in 3 or more autism siblings and that were found to be significantly differentially regulated in three independent mRNA expression experiments, lending support to the hypothesis of common molecular mechanisms between autism and other comorbid disorders.

## Discussion

In this study, we conducted a comparative analysis of autism and 31 comorbid conditions mined and retrieved from bibliome. By focusing on a set of 13 disorders that appeared to be most closely related to ASD (autism sibling comorbid conditions, see Fig. 1), we discovered that more than half of the autism genes included in our ASD seed list are also associated to related comorbid conditions. This finding supports our hypothesis, confirming the existence of molecular overlap and suggesting that these autism sibling comorbid disorders may share molecular mechanisms that could be enlightening for our understanding of the genetic etiology of ASD. Moreover, the multi-disorder component of the autism network (MDAG) is highly interconnected and significantly enriched for relevant and informative biological processes, such as synaptic transmission, neuron development, axonogenesis, transmission of nerve impulse and learning or memory, among others.

Motivated by these findings, we devised two analytical approaches to verify whether information from concurrent conditions could yield meaningful focus to the genome-wide search for ASD gene candidates. Our first approach, a process-based strategy, was grounded on the premise that processes for which the MDAG genes were enriched are generally relevant for neurological dysfunction. It is further predicated on the assumption that genes implicated in these processes that have been tied to one or more autism sibling comorbid disorders, but still have not yet been associated to ASD, should be autism gene candidates. To test this hypothesis we used available whole-genomic expression data from three independent experiments and found that 80 genes from our process-based candidate list were under significant differential expression in individuals with autism in the three datasets. The fact that they have been linked to neurological dysfunction together with having been implicated in biological processes that seem to play a role in autism makes these genes appealing new leads that may shed light in elucidating the molecular pathology of ASD.

The second approach, a network-based strategy, was based on the mainstream conception that protein interaction networks could give relevant and sometimes fortuitous leads for disease causative agents, suggesting potential points for biomarkers or drug targets and helping in elucidating the biological mechanisms involved [44–52]. In our approach, instead of looking at the whole protein interaction network, we took the set of all genes directly interacting with MDAG genes such that they contained only those proteins present in the list of autism sibling comorbid conditions, but absent from our seed list of published ASD candidates. Several genes within these network-based candidates have been previously related to neurological dysfunction. For instance, rare genetic variation in SLC1A2, necessary for proper synaptic activation and neurotransmission, has been associated with a wide range of neurological conditions including bipolar disorder, schizophrenia and autism [53]. Methylation of the glucocorticoid receptor gene NR3C1 through epigenetic processes, crucial in the hypothalamic-pituitary-adrenal axis modulation, our primary stress response system, has been linked to psychopathological conditions such as anxiety and depression [54, 55]. Variations in MAGI2, a synaptic scaffolding molecule with an essential role in synaptic transmission, are known to be related to epilepsy and cognitive impairment in patients with schizophrenia [56, 57]. In addition, mutations in CTNND2, a gene that plays a key role in neuronal development, particularly in the formation and maintenance of dendritic spines and synapses, have also been recently associated to autism [58]. Moreover, other candidate genes such as GRIA1, GRIA2, GABBR1, GABRG2, GABRR2, NRG2, NRG3, GRIK1, GRIK4, GRIN3A and GRM3, with functions that comprise formation of synapse, transmission of nerve impulse, behavior, learning or memory, are among families of genes that have been shown to have roles in neurological dysfunction jointly impacted in disorders like autism, schizophrenia and bipolar disorder [59–64]. Overall, the network-driven strategy yielded 91 genes found to be differentially expressed in individuals with autism when compared to healthy controls in all three experiments. This approach revealed the existence of a significant signal in the protein interaction networks of these related comorbid conditions, even one step removed from those genes that are shared among them (the MDAG). Even though these variations may represent real mechanistic differences between ASD and its sibling comorbid conditions, the overlap of 91 candidates found to be differentially regulated in autistic individuals from three independent datasets makes more likely that at least some reflect key holes in our understanding of autism.

In both analytical approaches, we were able to leverage the prior knowledge from two different sources, in this case from biological processes and protein interaction networks, to provide focused sets of candidates hypothesized to be under differential regulation in individuals with autism. From a methodological point of view, it is worth highlighting that in the absence of such prior knowledge several of the genes measured in the autistic patients included in all the three experiments would have had false discovery rate (FDR) values above the 0.05 threshold. In fact, this is a common circumstance in cases of weak signals and large background noise in several transcriptome-level experiments [65–67]. Conversely, with the utilization of prior knowledge, the major part of the candidate genes tested showed an FDR < 0.05. This turnabout of the frequent specificity problem at the genome-scale points towards a promising merging between knowledge and data-driven methodologies.

Finally, although the results evaluated herein should be considered preliminary, how the related comorbid disorder networks overlap with ASD have proved to be useful in enlightening important disease related biological processes and discovering potential autism candidate genes. In this work, by combining our process and network-based strategies, we were able to algorithmically assemble 19 candidate genes confirmed to be significantly differentially expressed in individuals with autism  from three independent experiments. Moreover, to better understand the biological significance of our final set of candidates, we tested their enrichment in signaling pathways and specific biological processes and whether they were interconnected within a biological network. Our analysis revealed that our predicted genes were implicated in 4 canonical pathways associated with brain structure and functioning, neuroinflammation, neurodegeneration, cognition and behavior [68–77]; alteration in these signaling pathways may play an important role in the pathophysiology of ASD.

Fourteen of these candidates interact with other molecules conforming a network significantly enriched in relevant biological processes related to normal brain growth and development. Dysregulation of any of these candidates may cause relevant disruptions in these fundamental processes altering neural outcomes and affecting cognition, learning and memory, especially since many of them interact with genes already associated to autism (APP, CYP19A1, ESR1, MAPK1, SETD2, SHANK2, TRPV1). In addition, some of the most connected nodes within the network (ESR1, TP53, AKT1, MAPK1, Pkcs, EGFR and APP) may support molecular mechanisms implicated in neuronal connectivity and synaptic plasticity; dysfunction in these neurological pathways have been linked to social and anxiety-related behaviors, mood conditions, cognitive degeneration and loss of neurological function, characteristic features observed in many neurological conditions, including ASD [78–90]. Finally, a remarkable connection was observed in this network between genes associated to ASD and other comorbid disorders and Luteinizing hormone (Lh), an endocrine hormone of the hypothalamic-pituitary-gonadal axis that acts in synergy with follicle-stimulating hormone (FSH), with roles in brain development and neuron differentiation [91]. Moreover, the regulation of these hormones release in blood is controlled by oxytocin, a neurohypophysial hormone that also operates as a brain neurotransmitter and that have been implicated in social behavior, recognition and bonding [92–96] and, therefore, alterations of its neuromodulatory activity have been associated to several mental disorders including autism [97–100]. Interestingly, dysregulation of the endocrine activity, particularly an interaction between potential ASD candidate genes and endocrine hormones of the hypothalamic-pituitary-gonadal axis was also found in our previous study [101]. Overall, these results lend additional support to the hypothesis that prior knowledge leveraged from comorbid conditions may contribute significantly to the progress in the genome wide search for autism candidate genes.

## Conclusion

A number of large-scale clinical studies have shown the high rates of comorbidity linked to autism that suggest the existence of an overlap in genes and biological processes in common between ASD and its co-occurring conditions. In the present work, we used a twofold systems biology approach to conduct a comparative analysis of autism and 31 comorbid disorders, with the aim of using the prior knowledge from these related conditions to predict 19 novel ASD gene candidates validated through transcriptome expression profiling experiments. This new set of genes appeared to be of potential etiologic relevance to ASD, as most of its members have been implicated in neurological processes critical for optimal brain growth and function, and have confirmed roles in neurological disease. Future work, including the evaluation of more comorbid conditions clustered in Fig. 1 and disorders neurological in nature or nor (for instance, autoimmune disorders), may be useful in the effort to arrange and reorder genes that have been associated to autism so far, and possibly unveil new genes worth investigating for our understanding of the pathophysiology of autism.

## Additional files

Additional file 1: Table S1. Information summary of the datasets selected. (DOCX 12 kb)

Additional file 2: Figure S1. Box plots showing the distribution of the samples of each dataset after preprocessing; median-centered values indicate that the data are normalized and cross-comparable. (PDF 5181 kb)

Additional file 3: Table S2. Complete list of comorbid conditions to autism. Autism sibling disorders are highlighted in blue. (XLSX 16 kb)

Additional file 4: Table S3. Total number of genes of each comorbid condition utilized for this study. Highlighted in blue are ASD sibling disorders. (XLSX 135 kb)

Additional file 5: Table S4. Comorbid disorders integrating each group generated by the bootstrap analysis (Additional file 6: Figure S2), along with their Mean Jaccard Coefficient value. The different groups of disorders generated by our bootstrap procedure corresponds to the disorder clusters obtained in our original gene-based dendrogram (Fig. 1). Groups 1, 2 and 3 have the highest Mean Jaccard values meaning they are the most robust and stable groupings of the tree. Group 2 coincides with the cluster conformed by the autism sibling disorders with a highly significant Mean Jaccard value of approximately 0.785. (DOCX 13 kb)

Additional file 6: Figure S2. First two Multidimensional Scaling (MDS) dimensions of our dataset generated by MDS on a dissimilarity matrix using Jaccard Coefficient when k = 6. Each group is highlighted in a different color and the disorders conforming them are detailed in Additional file 5: Table S4, along with their corresponding mean Jaccard Coefficient value. The autism sibling comorbid disorders are clustered together in group 2 (PDF 180 kb)

Additional file 7: Table S5. STRING edge summary for each member of the ASD sibling group. (XLSX 8411 kb)

Additional file 8: Table S6. The multi disorder autism gene set (MDAG) and the sibling comorbid conditions where these genes are found. (XLSX 25 kb)

Additional file 9: Figure S3. A. The complete network of autism candidate genes. The MDAG genes are highlighted in yellow and their interactions in red; these are the genes that occur in one or more of the autism sibling comorbid disorders, circumscribed in Fig. 1. B. The highly interconnected subcomponent conformed by the MDAG genes, separated from the autism network. (TIF 406700 kb)

Additional file 10: Table S7. Biological processes for which the Multi-disorder component of the autism gene set (MDAG) were enriched. Identities of the MDAG genes overrepresented in the processes as well as the corrected *p*-values for the enrichment scores are provided. Enrichment was calculated using the biological processes only found among the MDAG genes and not found among the sibling disorders. (XLSX 50 kb)

Additional file 11: Table S8. Identities of the differentially expressed PBC, along with their corresponding *q*-values, the biological processes where they are involved and the comorbid disorders where they are implicated. Also, PBC significantly differentially expressed in each dataset and in all the three datasets. (XLSX 210 kb)

Additional file 12: Figure S4. *P*-value and *q*-value histograms and q-plots from the multiple test correction analyses performed on the PBC and NBC to verify whether they were significantly differentially regulated in autistics in comparison to controls. (PDF 11411 kb)

Additional file 13: Table S9. Complete list of NBC, along with their *q*-values, MDAG interactors and comorbid disorders where they are present. Also, NBC significantly differentially expressed in each dataset and in all the three datasets. (XLSX 185 kb)

Additional file 14: Table S10. Complete list of genes present in the intersection of PBC∩NBC, along with the biological processes where they are involved, MDAG interactors and comorbid disorders where they are present. Also, PBC∩NBC significantly differentially expressed in each dataset and in all the three datasets. (XLSX 226 kb)

## Abbreviations

ADHD: Attention Deficit Hyperactivity Disorder
ASD: Autism Spectrum Disorder
BP: Biological Processes
CDC: Center for Disease Control and Prevention
DAVID: Database for Annotation, Visualization and Integrated Discovery
FDR: False Discovery Rate
FSH: Follicle-Stimulating Hormone
GEO: Gene Expression Omnibus
GO: Gene Ontology
ICD-9: International Classification of Diseases, 9th version
IPA®: Ingenuity® Pathway Analysis software
KEGG: Kyoto Encyclopedia of Genes and Genomes
Lh: Luteinizing hormone
MATLAB®: MATrix LABoratory software
MDAG: Multi Disorder Autism Gene Set
MeSH: Medical Subject Headings
mRNA: Messenger Ribonucleic Acid
NBC: Network-based Candidates
NCHS: National Center for Health Statistics
PBC: Process-based Candidates
RMA: Robust Multiarray Average
RNA: Ribonucleic acid
SFARI: Simons Foundation Autism Research Initiative
STRING: Search Tool for the Retrieval of Interacting Genes/Proteins
