# Supplementary material for: Cross-disorder comparative analysis of comorbid conditions reveals novel autism candidate genes
Source: BMC Genomics. 2017 Apr 20;18:315. doi: 10.1186/s12864-017-3667-9 (PMC5399393; doi:10.1186/s12864-017-3667-9)
Supplement: Supplementary file 1 — Information summary of the datasets selected. (DOCX 12 kb) [file 12864_2017_3667_MOESM1_ESM.docx]

| **Dataset** | **Platform** | **Data Format** | **# samples** | **# individuals** | **Gender** | **Mean Age (ASD/control)** |
| --- | --- | --- | --- | --- | --- | --- |
| **GSE18123**  (2012) | GPL570 | CEL files  (raw data) | 66 ASD (31 autism, 26 PDDNOS, 9 Asperger)  33 Controls | 66 ASD  33 controls | male | 8/9 |
| **GSE25507**  (2011) | GPL570 | CEL Files  (raw data) | 82 autism  64 controls | 82 autism  64 controls | male | 5.5/7.9 |
| **GSE42133**  (2015) | GPL10558 | Normalized data Series matrix (txt) | 91 ASD  56 controls | 87 ASD  55 controls | male | 2.3/2 |

Table S1. Information summary of the datasets selected.
