# Supplementary figures and images for: Cross-disorder comparative analysis of comorbid conditions reveals novel autism candidate genes
Source: BMC Genomics. 2017 Apr 20;18:315. doi: 10.1186/s12864-017-3667-9 (PMC5399393; doi:10.1186/s12864-017-3667-9)

# GSE18123gpl570

NUSE

RLE

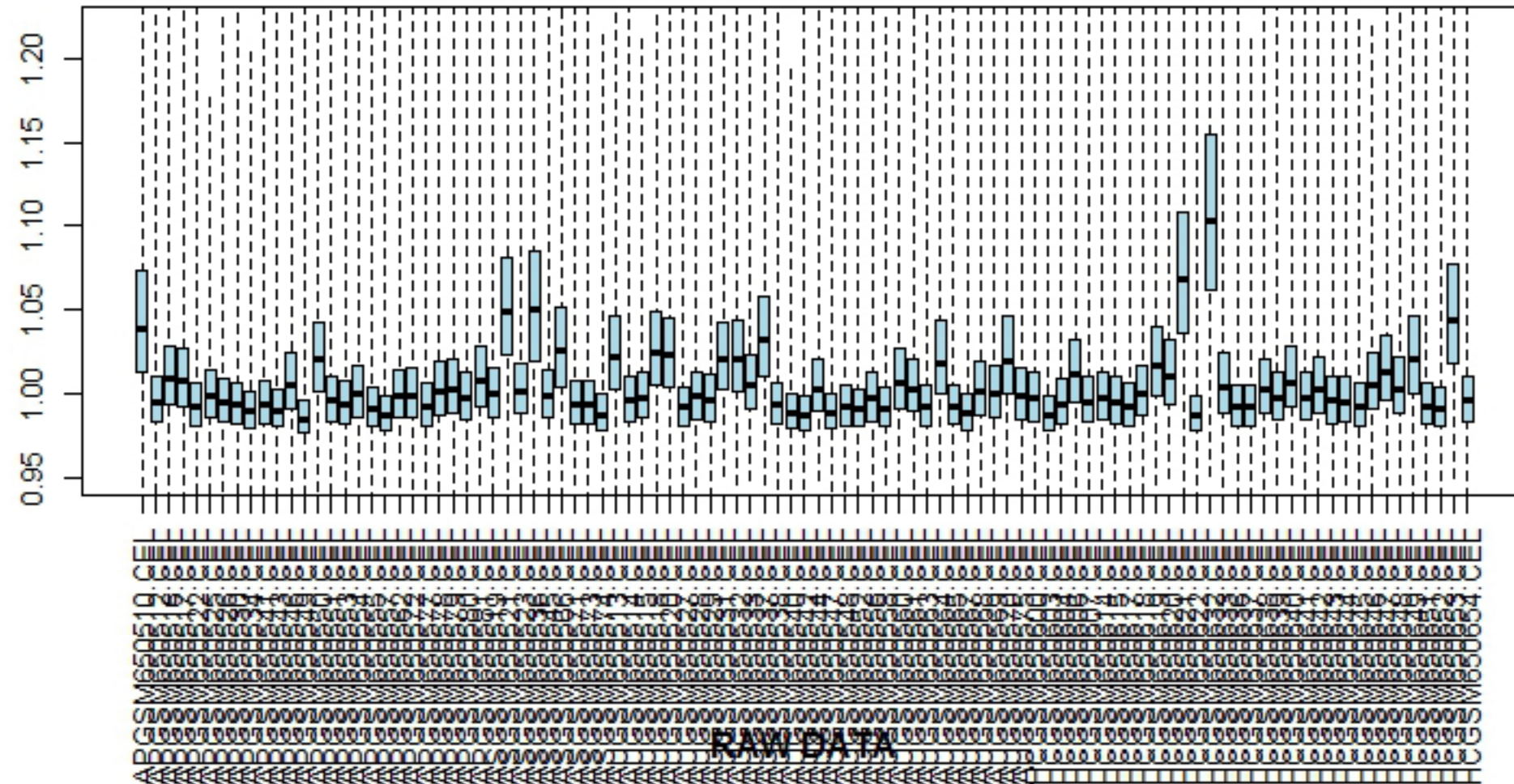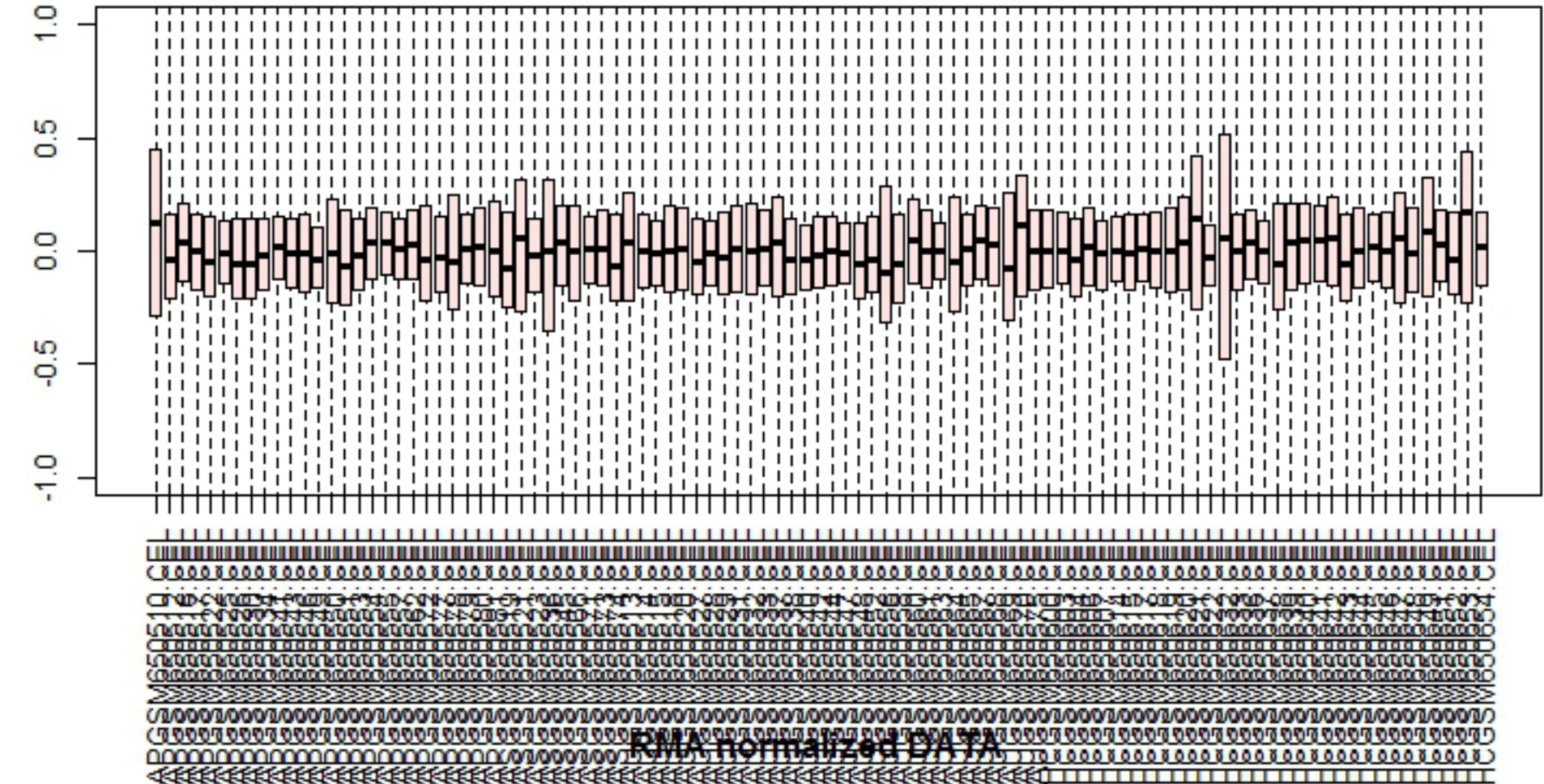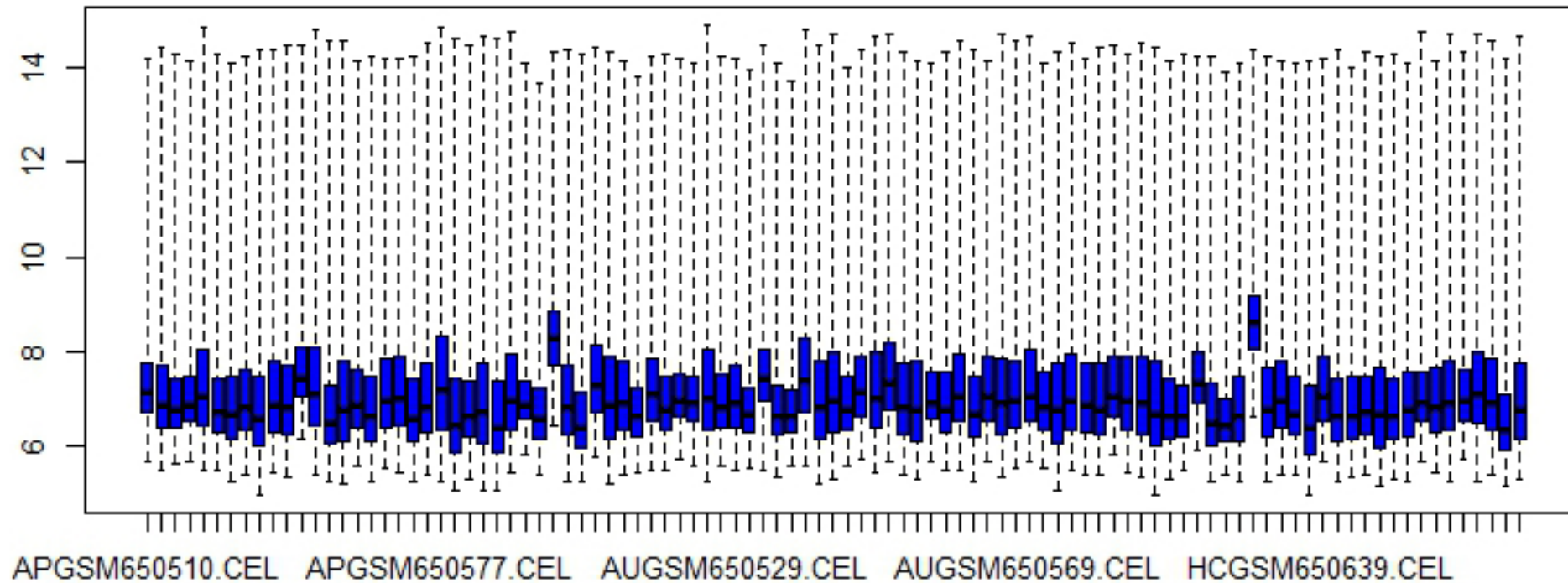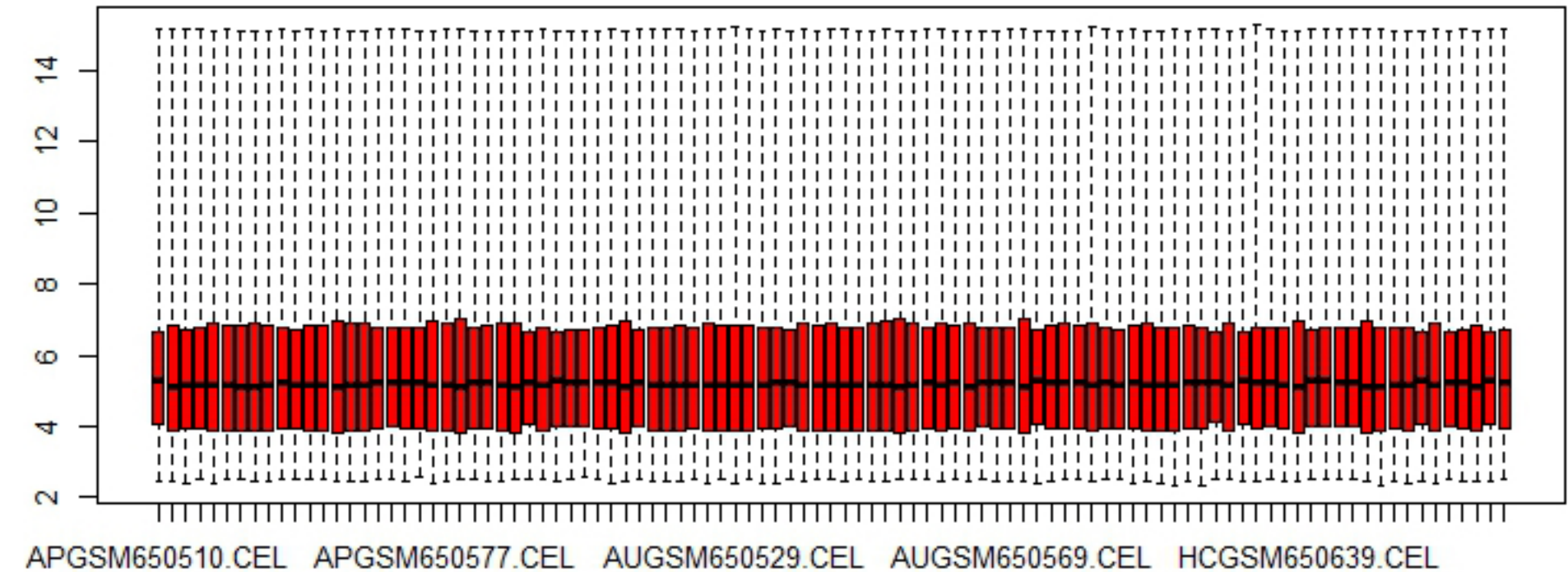

Raw data

RMA normalized data

GSE25507

NUSE

RLE

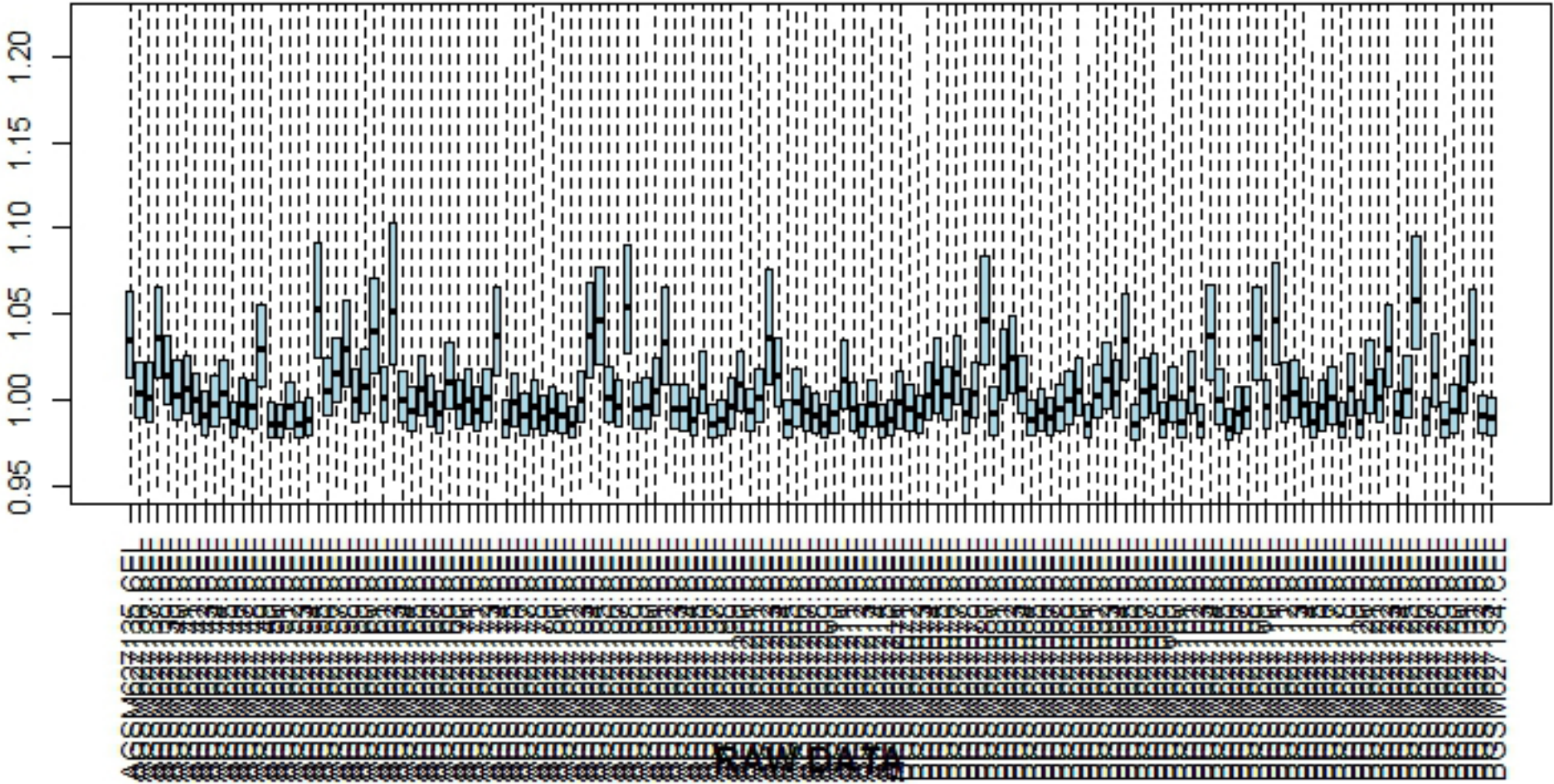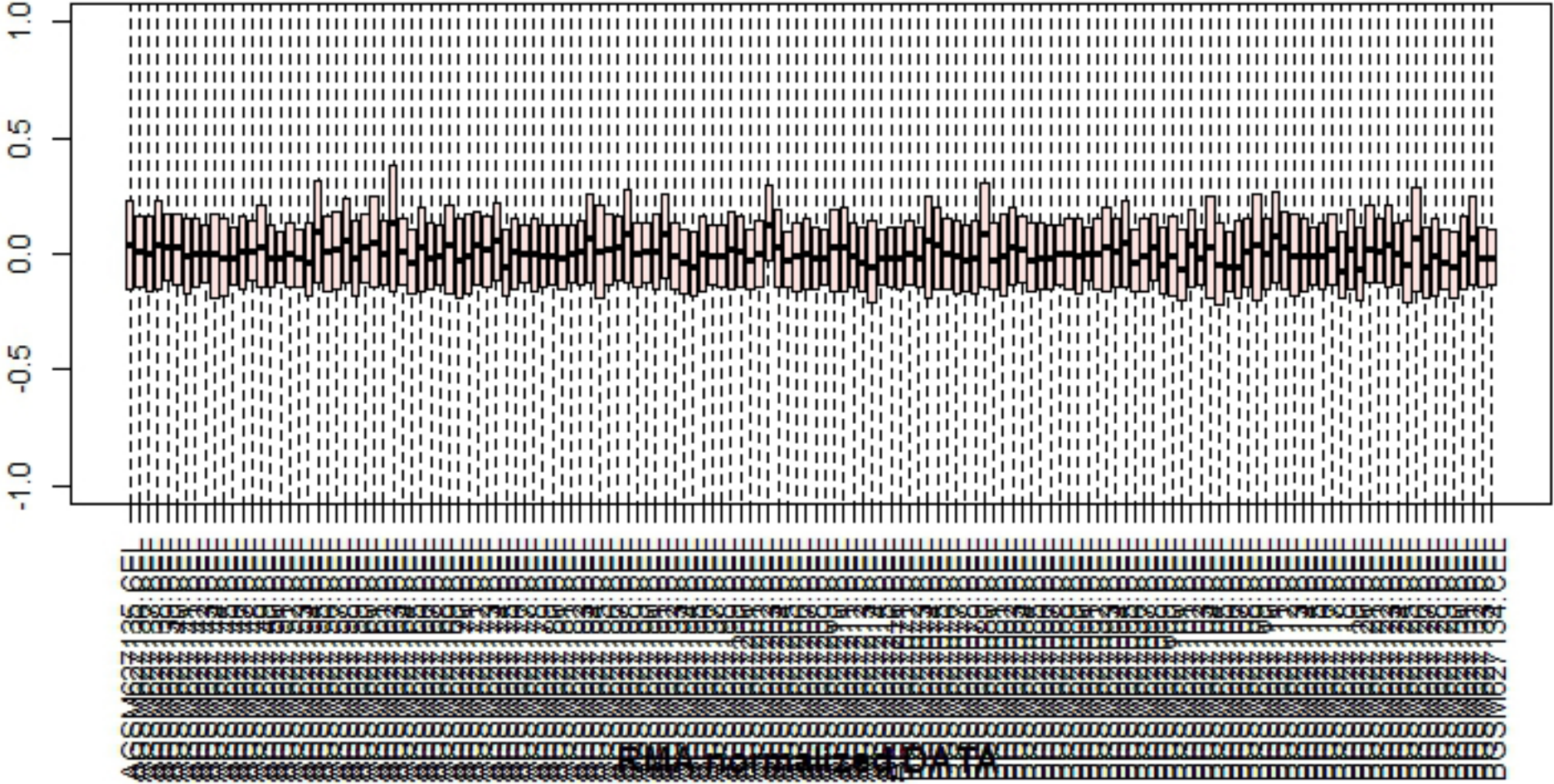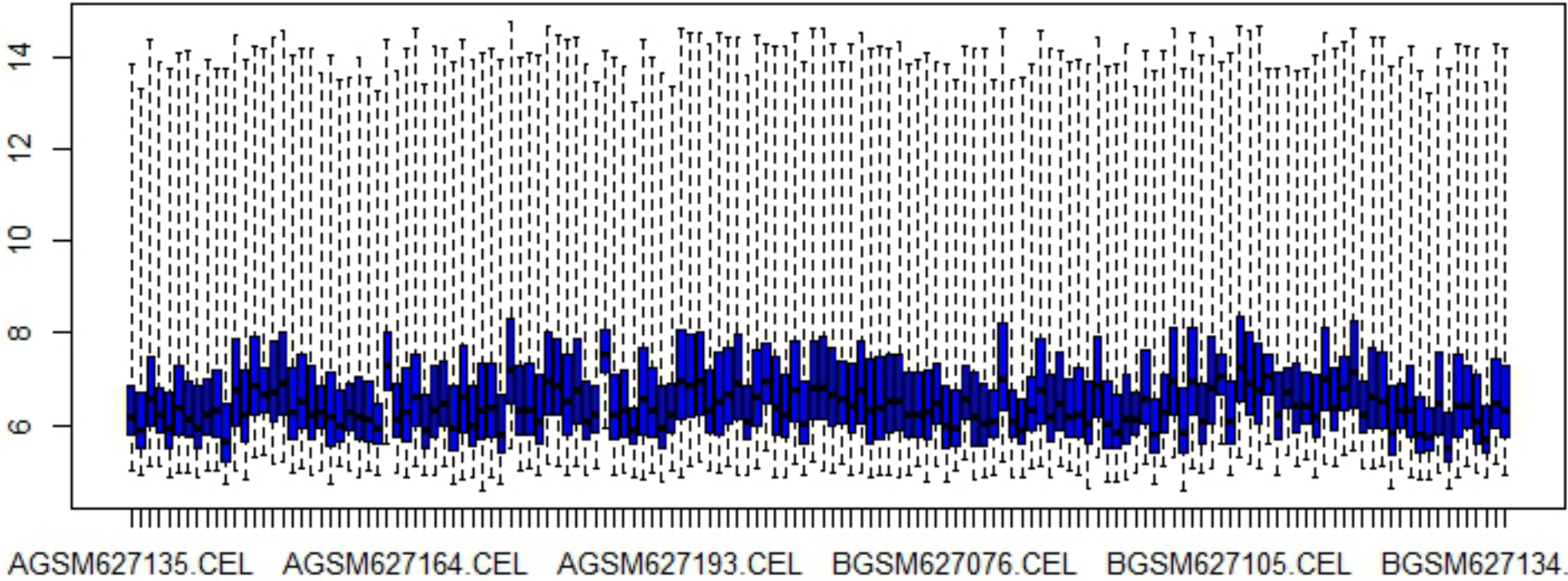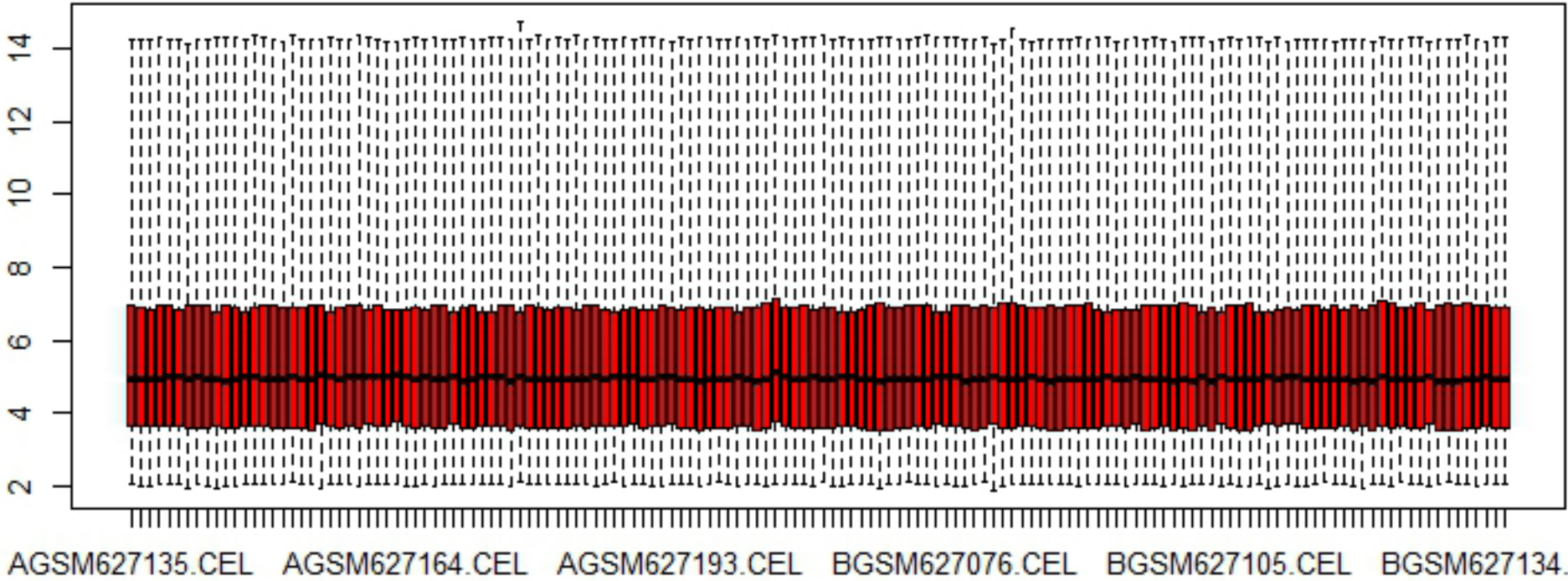

Raw data

RMA normalized data

GSE42133

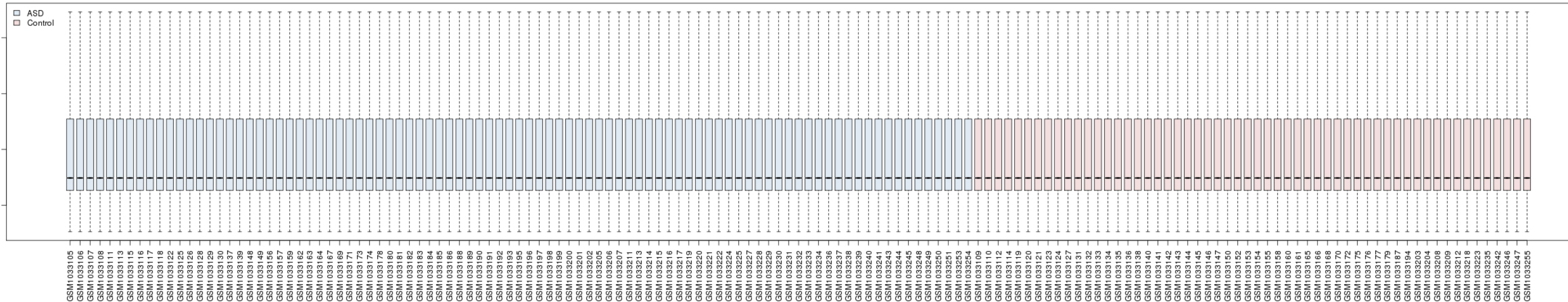

Supplement: Supplementary file 2 — Box plots showing the distribution of the samples of each dataset after preprocessing; median-centered values indicate that the data are normalized and cross-comparable. (PDF 5181 kb) [file 12864_2017_3667_MOESM2_ESM.pdf]
