# Supplementary material for: Cross-disorder comparative analysis of comorbid conditions reveals novel autism candidate genes
Source: BMC Genomics. 2017 Apr 20;18:315. doi: 10.1186/s12864-017-3667-9 (PMC5399393; doi:10.1186/s12864-017-3667-9)
Supplement: Supplementary file 5 — Comorbid disorders integrating each group generated by the bootstrap analysis (Additional file 6: Figure S2), along with their Mean Jaccard Coefficient value. The different groups of disorders generated by our bootstrap procedure corresponds to the disorder clusters obtained in our original gene-based dendrogram (Fig. 1). Groups 1, 2 and 3 have the highest Mean Jaccard values meaning they are the most robust and stable groupings of the tree. Group 2 coincides with the cluster conformed by the autism sibling disorders with a highly significant Mean Jaccard value of approximately 0.785. (DOCX 13 kb) [file 12864_2017_3667_MOESM5_ESM.docx]

| **Groups K=6** | **Comorbid Disorders** | **Mean Jaccard Coefficient** |
| --- | --- | --- |
| 1 | Agoraphobia, Communication Deficits, Conduct disorder, Developmental Coordination Disorder, Learning Disorders, Simple Phobia, Tic Disorders, Tourette Syndrome | 0.8157727 |
| 2 | Anxiety Disorder, Attention Deficit Hyperactivity Disorder, Autistic Disorder, Bipolar Spectrum Disorders, Depressive Disorder, Down syndrome, Epilepsy, Fragile X Syndrome, Intellectual Disability, Obsessive Compulsive Disorder, Panic Disorder, Schizophrenia, Sleep Disorders, Tuberous Sclerosis | 0.7847099 |
| 3 | Autoimmune Disorders, Bowel Disorder (non IBD), CNS Cranial Anomalies, Diabetes Mellitus Type 1, Imflammatory Bowel Disease, Immune Disorders, Sensory Problems | 0.8979880 |
| 4 | Encopresis | 0.5690881 |
| 5 | Enuresis | 0.6320000 |
| 6 | Muscular Dystrophy | 0.5921143 |

**Table S4**. Comorbid disorders integrating each group generated by the bootstrap analysis (Supplementary Figure S2), along with their Mean Jaccard Coefficient value. The different groups of disorders generated by our bootstrap procedure corresponds to the disorder clusters obtained in our original gene-based dendrogram (Figure 1). Groups 1, 2 and 3 have the highest Mean Jaccard values meaning they are the most robust and stable groupings of the tree. Group 2 coincides with the cluster conformed by the autism sibling disorders with a highly significant Mean Jaccard value of approximately 0.785.
