# Supplementary material for: Cross-disorder comparative analysis of comorbid conditions reveals novel autism candidate genes
Source: BMC Genomics. 2017 Apr 20;18:315. doi: 10.1186/s12864-017-3667-9 (PMC5399393; doi:10.1186/s12864-017-3667-9)
Supplement: Supplementary file 6 — First two Multidimensional Scaling (MDS) dimensions of our dataset generated by MDS on a dissimilarity matrix using Jaccard Coefficient when k = 6. Each group is highlighted in a different color and the disorders conforming them are detailed in Additional file 5: Table S4, along with their corresponding mean Jaccard Coefficient value. The autism sibling comorbid disorders are clustered together in group 2 (PDF 180 kb) [file 12864_2017_3667_MOESM6_ESM.pdf]

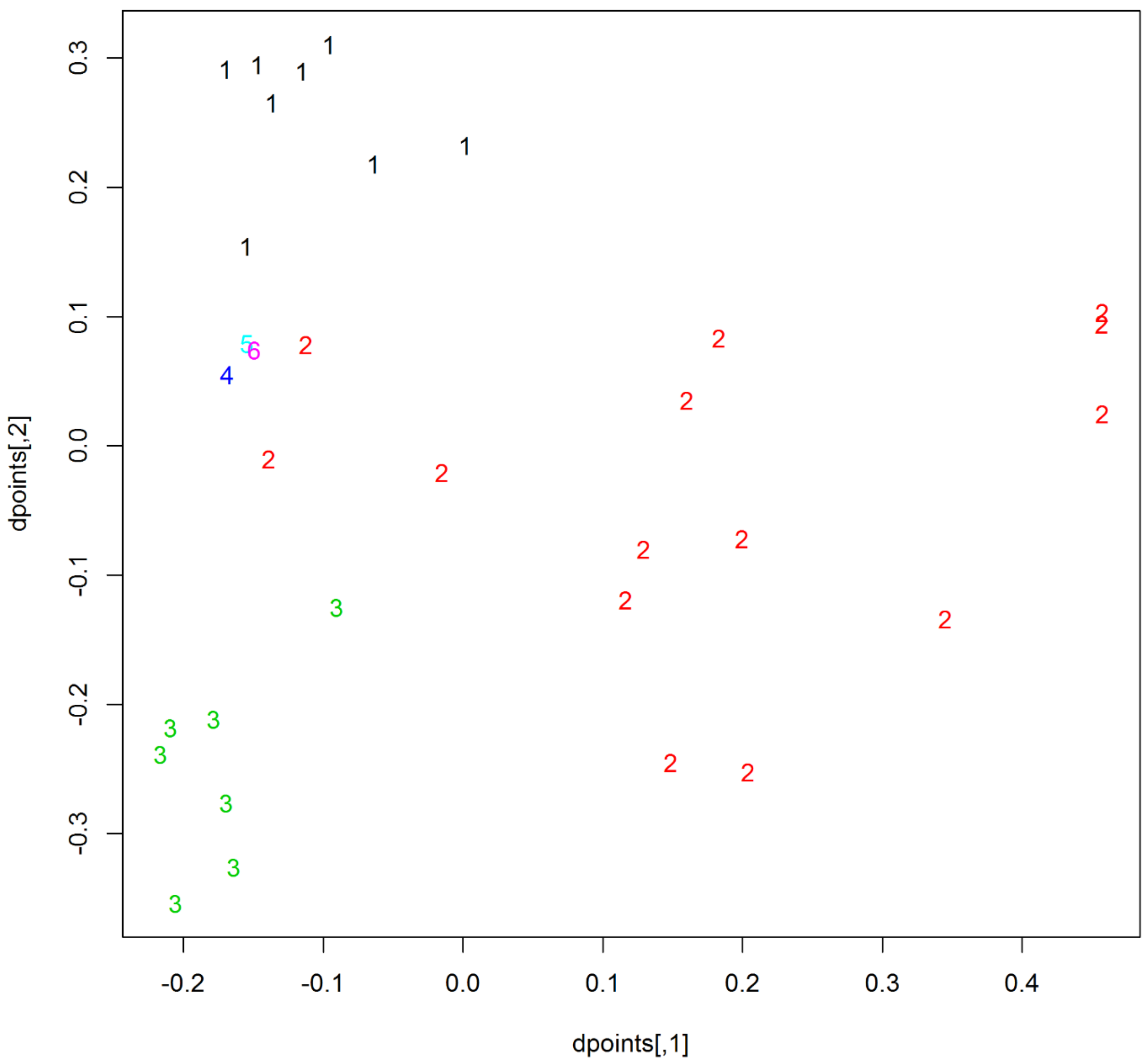

Figure S2. First two Multidimensional Scaling (MDS) dimensions of our dataset generated by MDS on a dissimilarity matrix using Jaccard Coefficient when k=6. Each group is highlighted in a different color and the disorders conforming them are detailed in Supplementary Table S4, along with their corresponding mean Jaccard Coefficient value. The autism sibling comorbid disorders are clustered together in group 2.
