# Supplementary material for: Cross-disorder comparative analysis of comorbid conditions reveals novel autism candidate genes
Source: BMC Genomics. 2017 Apr 20;18:315. doi: 10.1186/s12864-017-3667-9 (PMC5399393; doi:10.1186/s12864-017-3667-9)
Supplement: Supplementary file 12 — P-value and q-value histograms and q-plots from the multiple test correction analyses performed on the PBC and NBC to verify whether they were significantly differentially regulated in autistics in comparison to controls. (PDF 11411 kb) [file 12864_2017_3667_MOESM12_ESM.pdf]

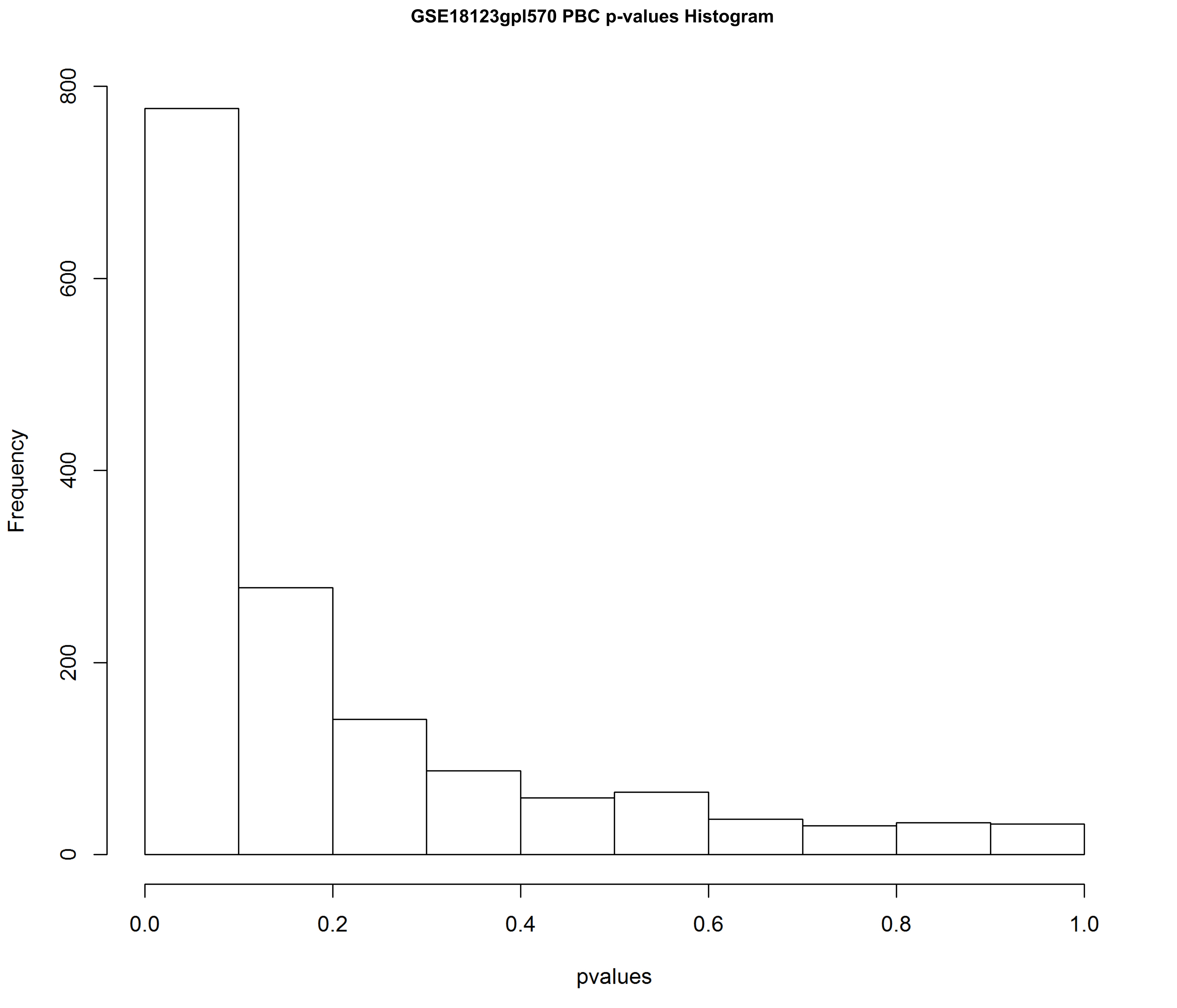

GSE18123gpl570 PBC p-values Histogram
Frequency
0
200
400
600
800
0.0
0.2
0.4
0.6
0.8
1.0
pvalues

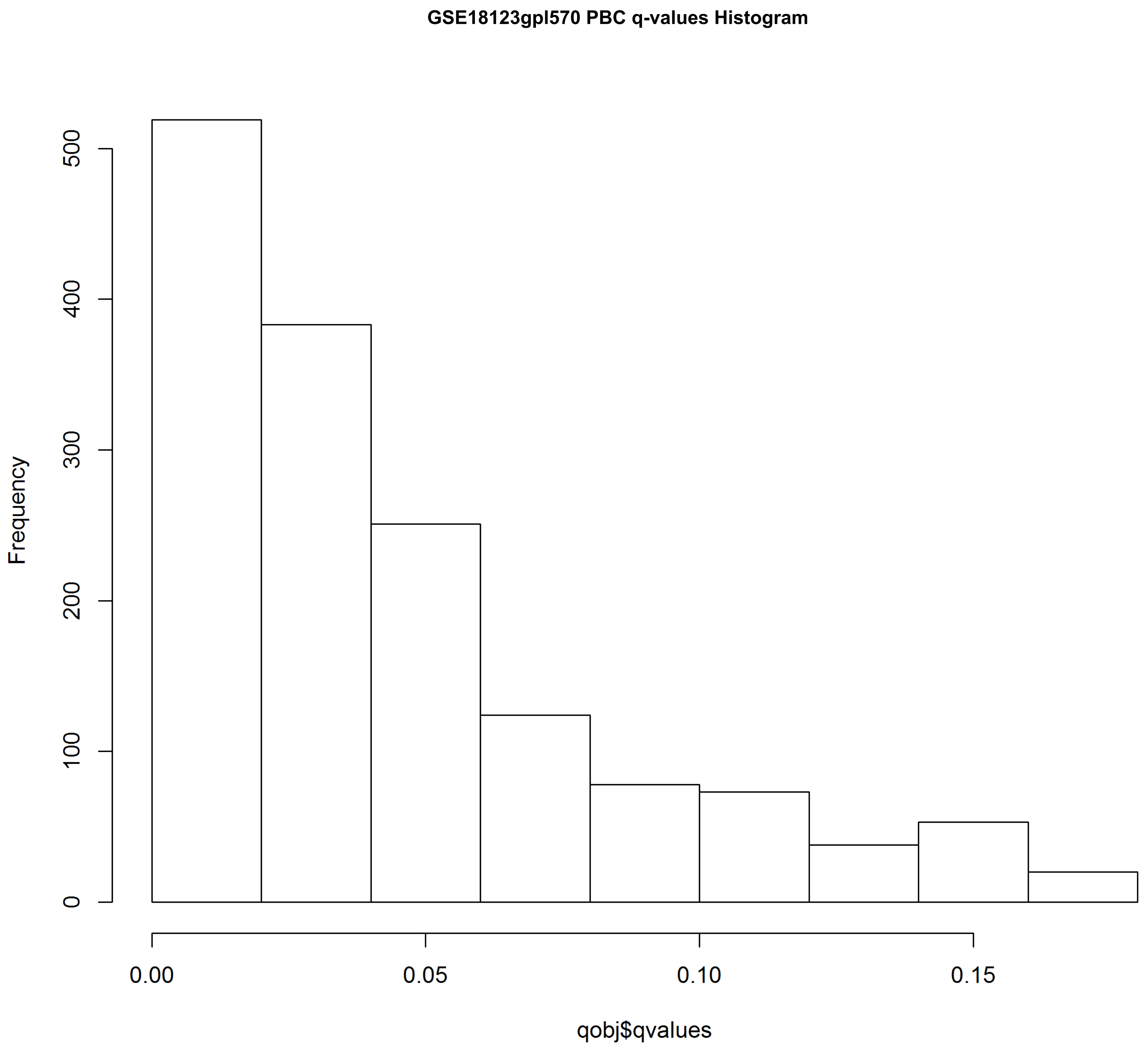
GSE18123gpl570 PBC q-values Histogram
Frequency
0
100
200
300
400
500
0.00
0.05
0.10
0.15
qobj$qvalues

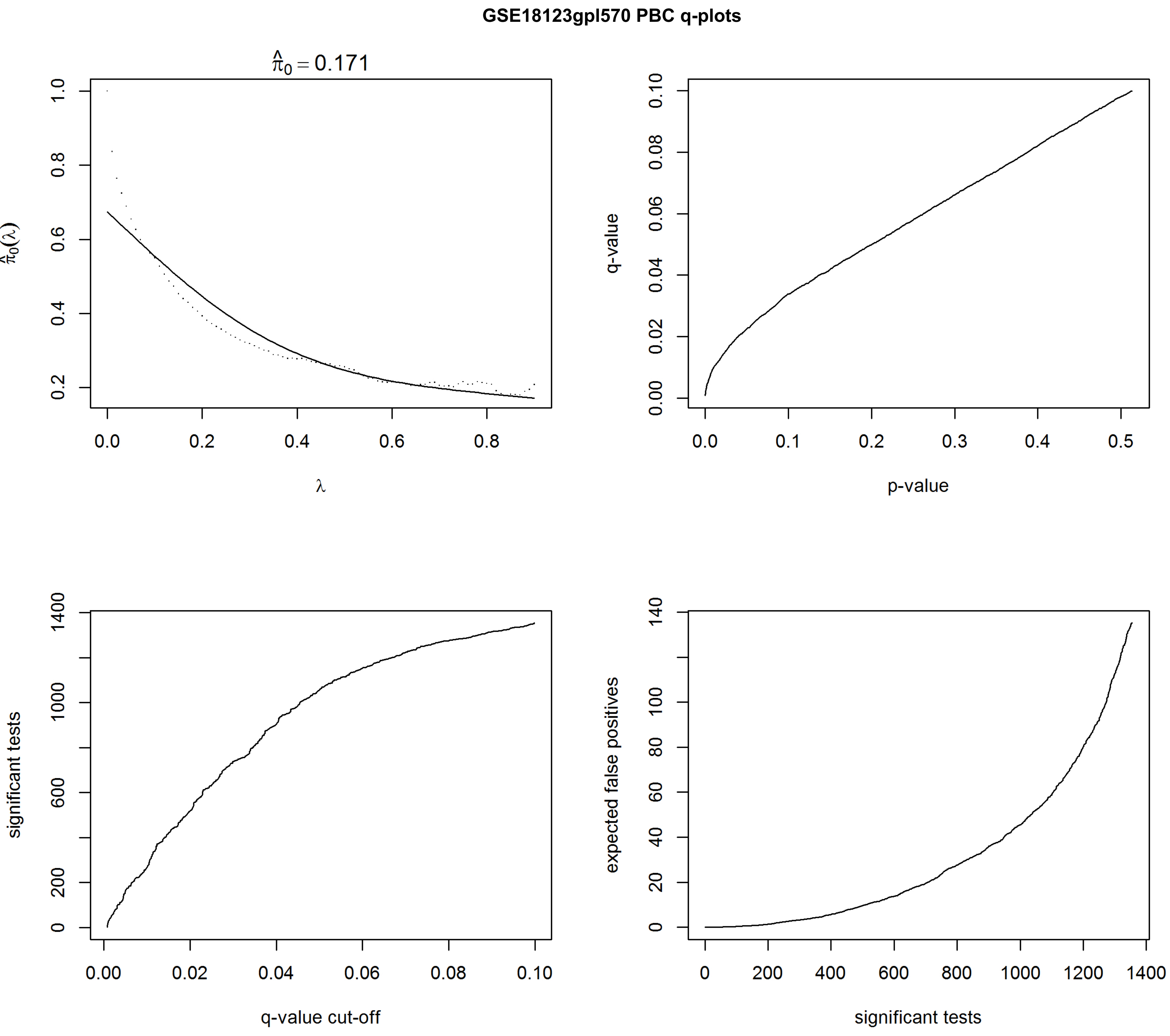
GSE18123gpl570 PBC q-plots
$\hat{\pi}_0 = 0.171$
$\hat{\pi}_0(\lambda)$
λ
q-value
p-value
significant tests
q-value cut-off
expected false positives
significant tests

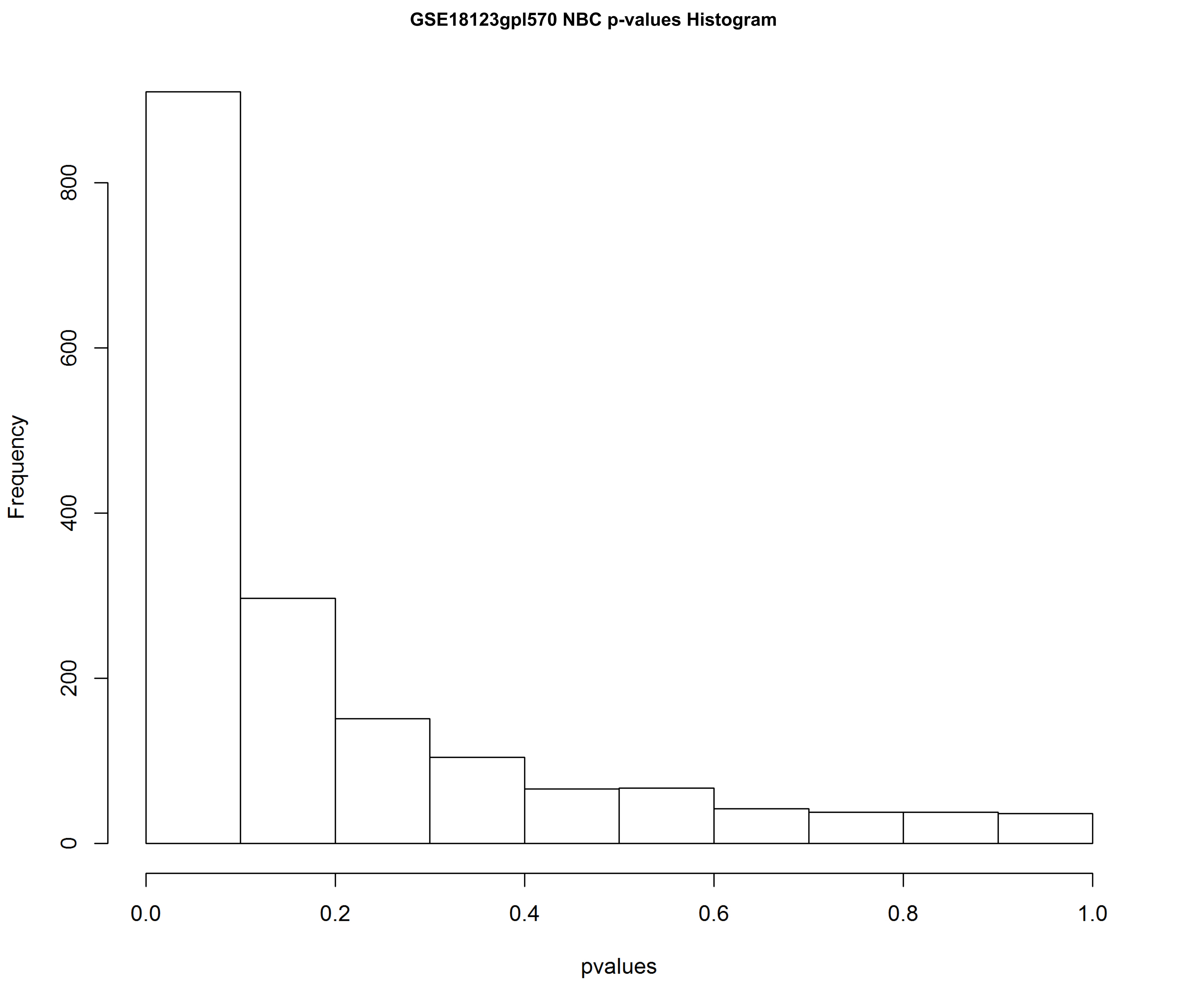
GSE18123gpl570 NBC p-values Histogram
Frequency
0
200
400
600
800
0.0
0.2
0.4
0.6
0.8
1.0
pvalues

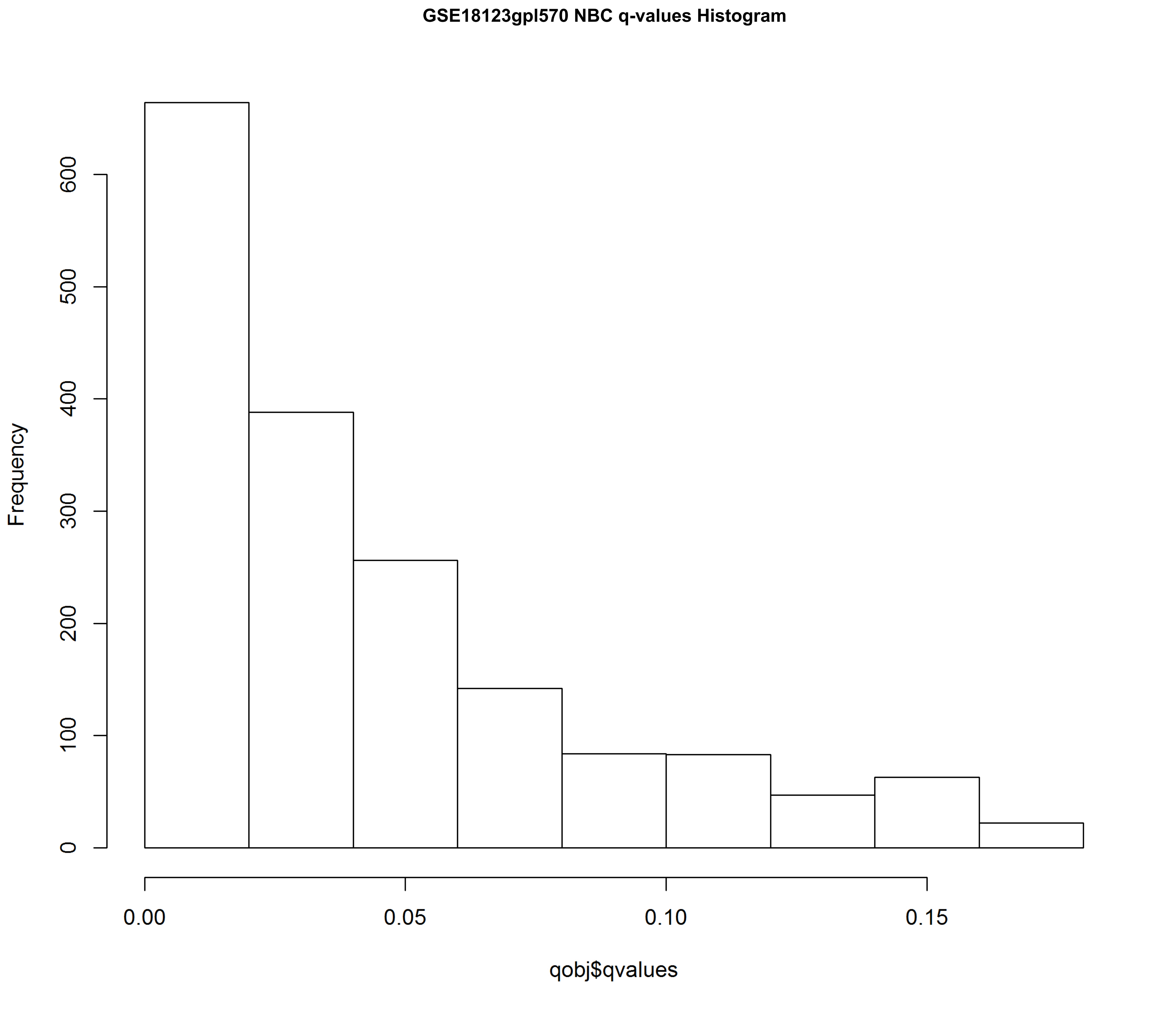
GSE18123gpl570 NBC q-values Histogram
Frequency
0
100
200
300
400
500
600
0.00
0.05
0.10
0.15
qobj$qvalues

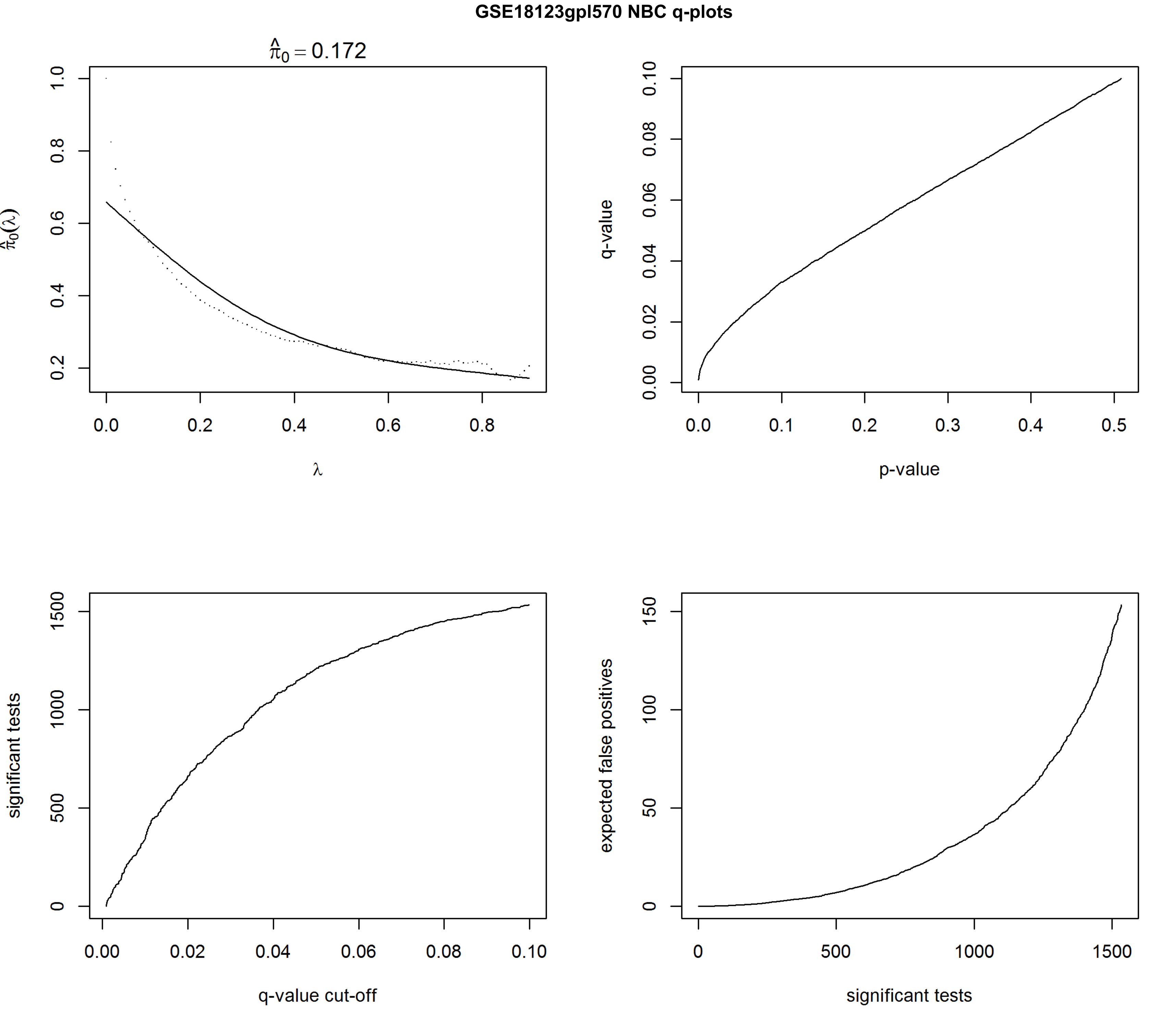

GSE18123gpl570 NBC q-plots
$\hat{\pi}_0 = 0.172$
$\hat{\pi}_0(\lambda)$
λ
q-value
p-value
significant tests
q-value cut-off
expected false positives
significant tests

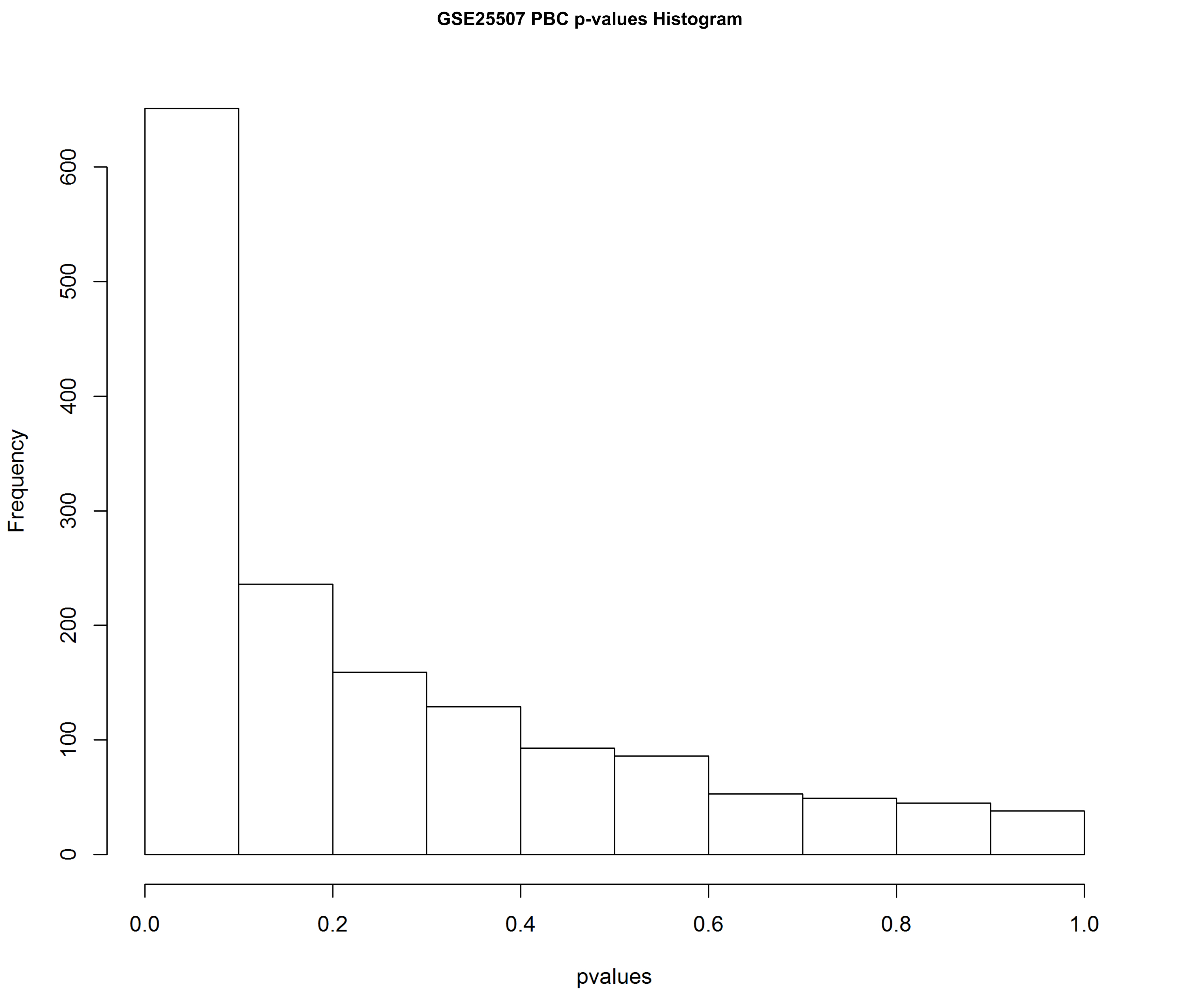
GSE25507 PBC p-values Histogram
Frequency
0
100
200
300
400
500
600
0.0
0.2
0.4
0.6
0.8
1.0
pvalues

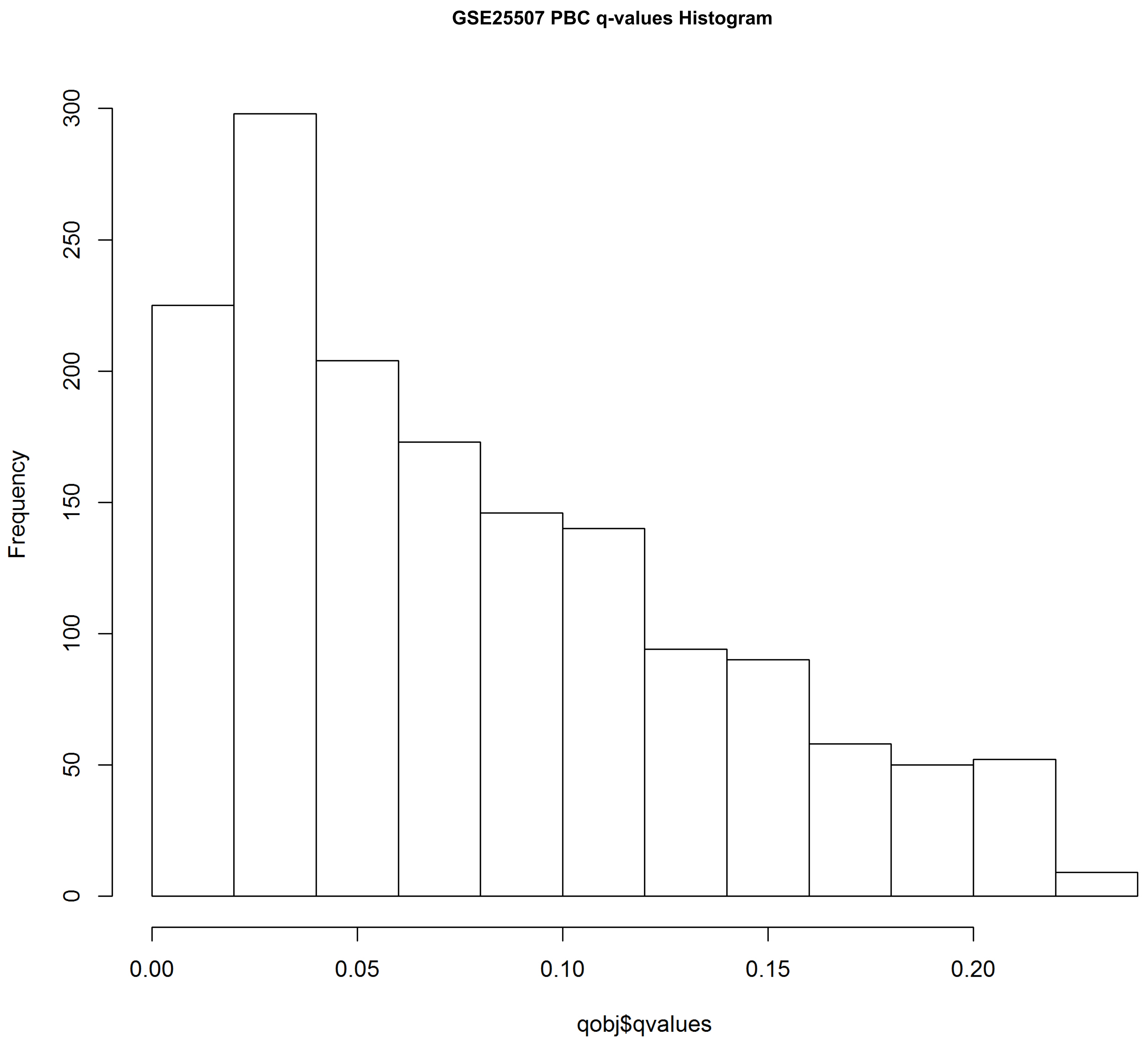
GSE25507 PBC q-values Histogram
Frequency
0
50
100
150
200
250
300
0.00
0.05
0.10
0.15
0.20
qobj$qvalues

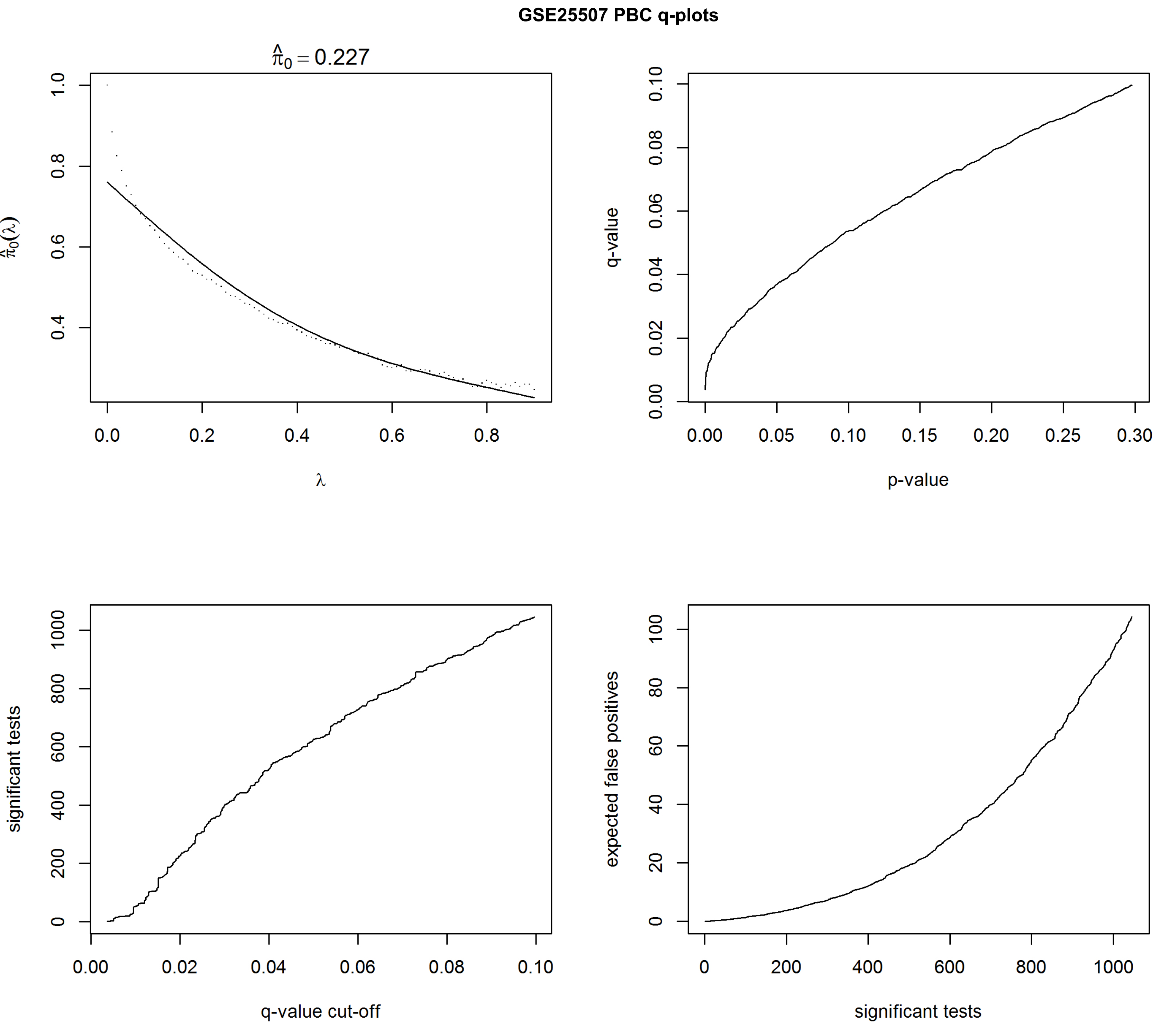

GSE25507 PBC q-plots
$\hat{\pi}_0 = 0.227$
$\hat{\pi}_0(\lambda)$
λ
q-value
p-value
significant tests
q-value cut-off
expected false positives
significant tests

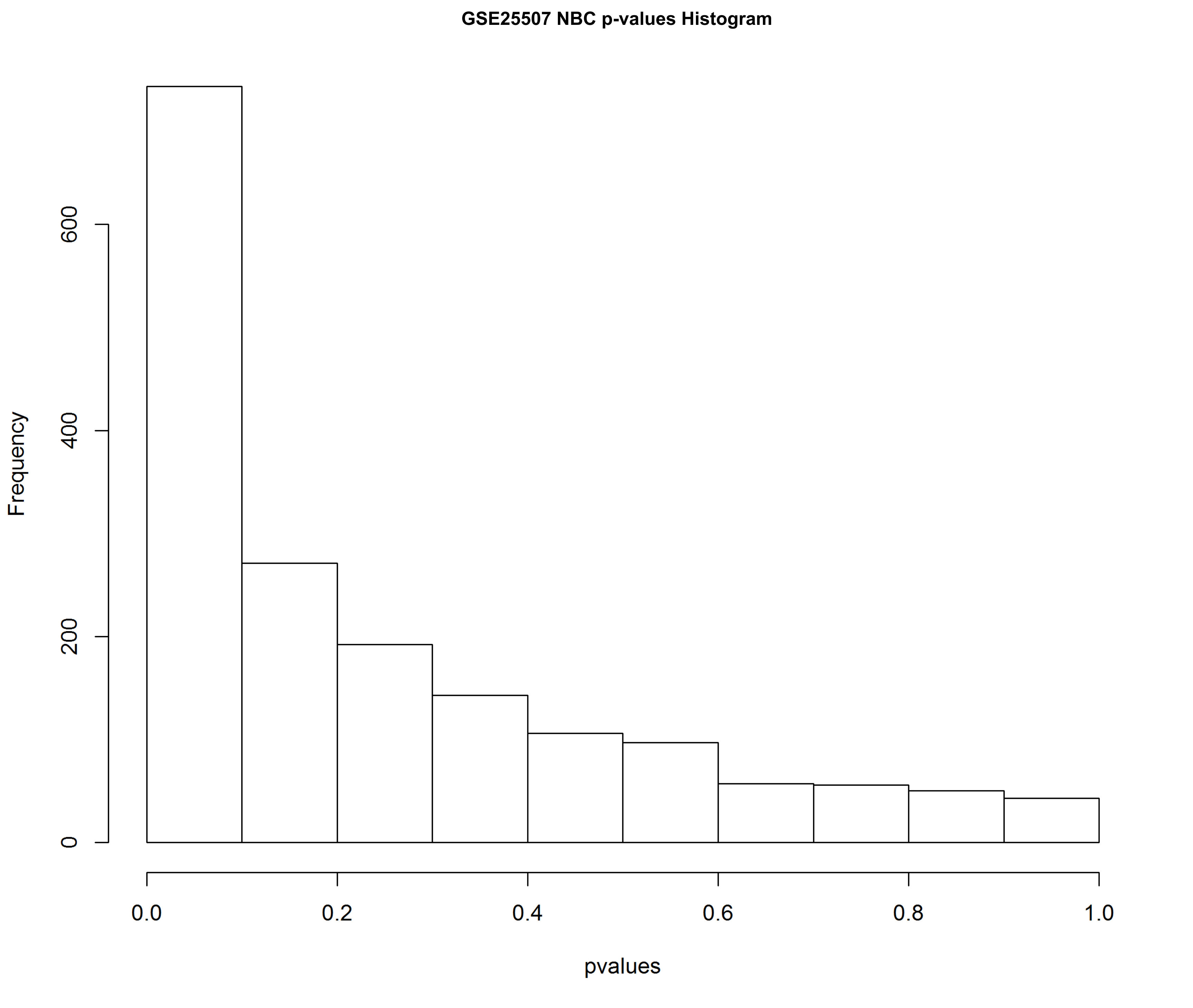
GSE25507 NBC p-values Histogram
Frequency
0
200
400
600
0.0
0.2
0.4
0.6
0.8
1.0
pvalues

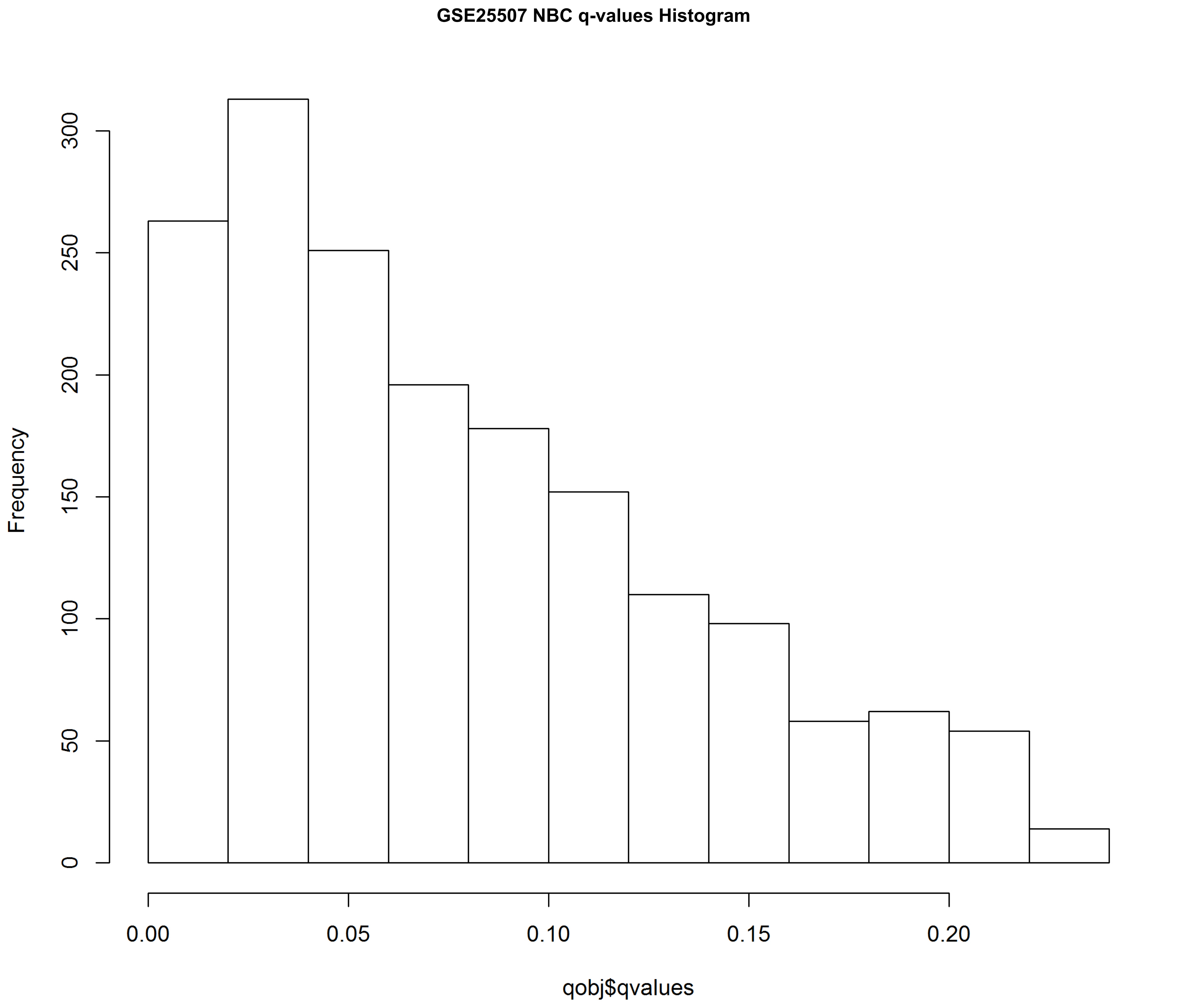

GSE25507 NBC q-values Histogram
Frequency
0
50
100
150
200
250
300
0.00
0.05
0.10
0.15
0.20
qobj$qvalues

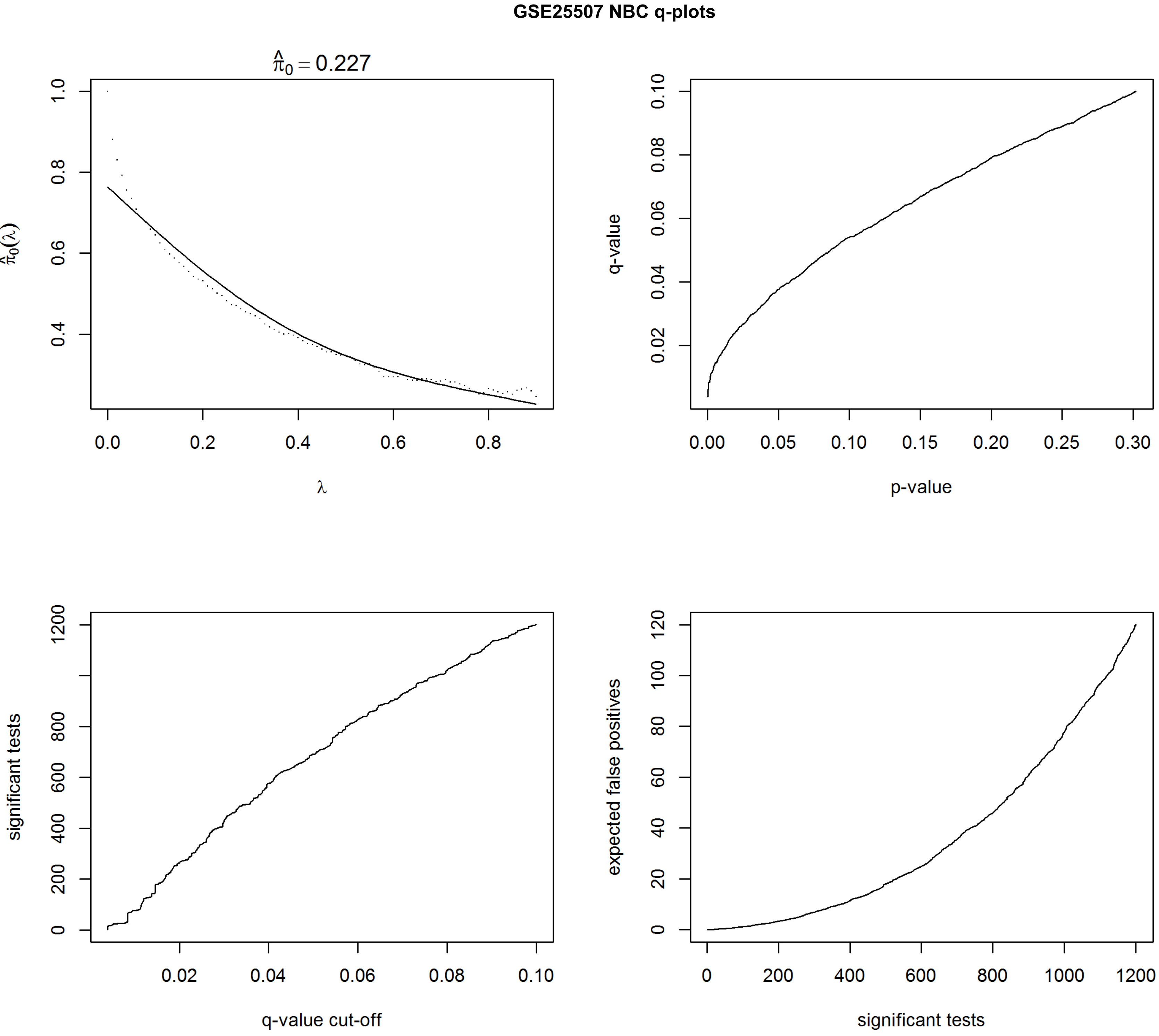
GSE25507 NBC q-plots
$\hat{\pi}_0 = 0.227$
$\hat{\pi}_0(\lambda)$
λ
q-value
p-value
significant tests
q-value cut-off
expected false positives
significant tests

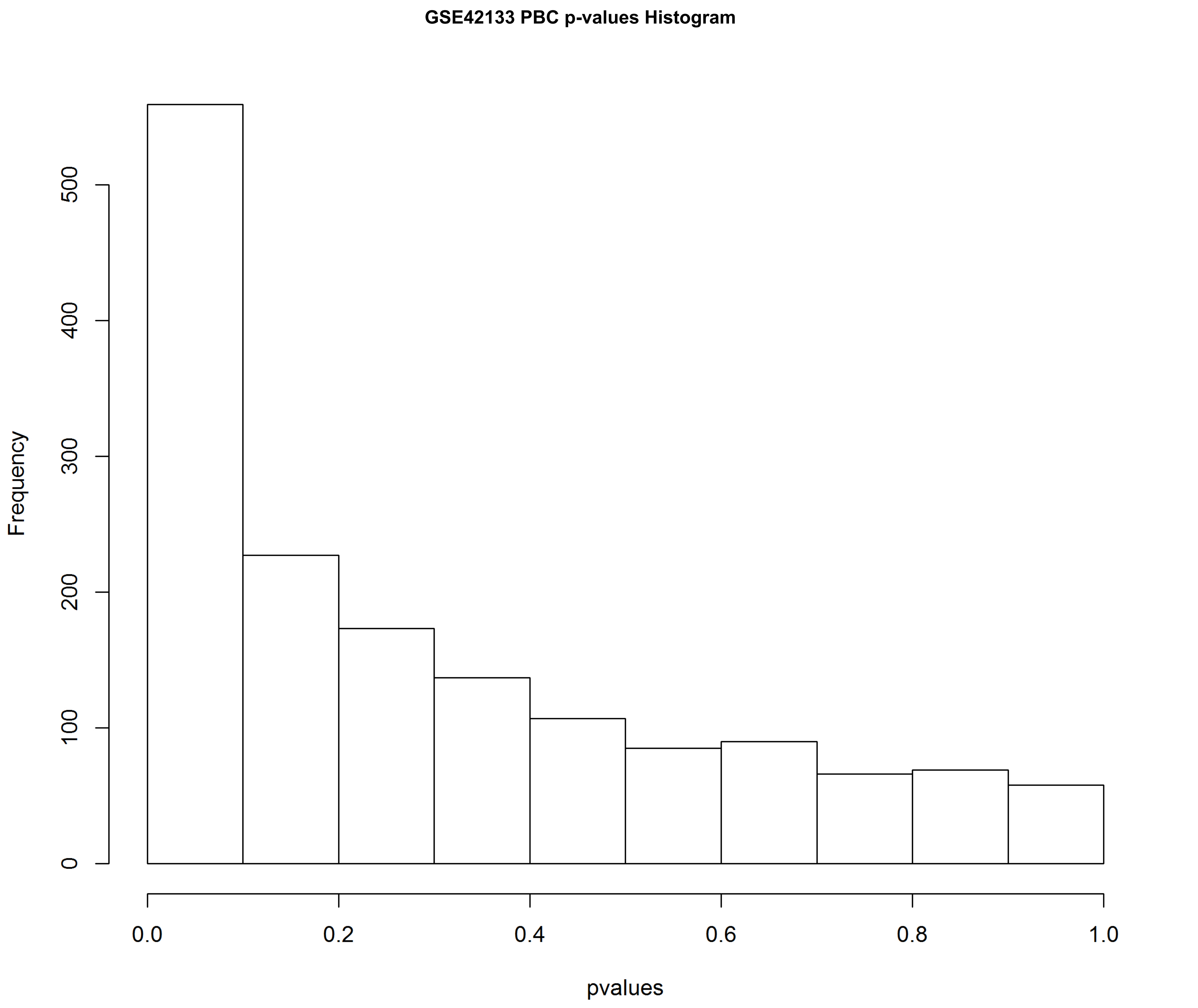
GSE42133 PBC p-values Histogram
Frequency
0
100
200
300
400
500
0.0
0.2
0.4
0.6
0.8
1.0
pvalues

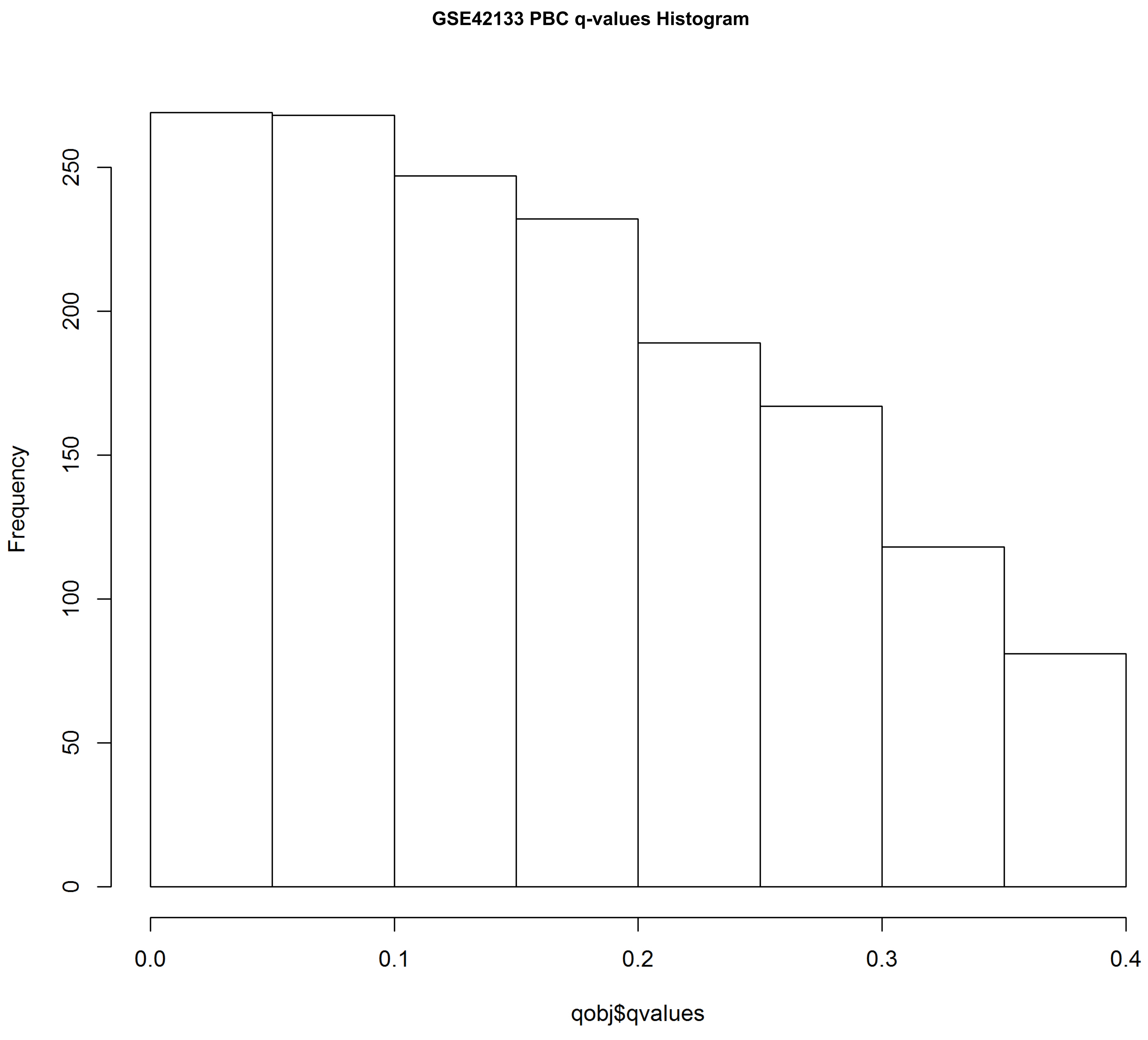
GSE42133 PBC q-values Histogram
Frequency
0
50
100
150
200
250
0.0
0.1
0.2
0.3
0.4
qobj$qvalues

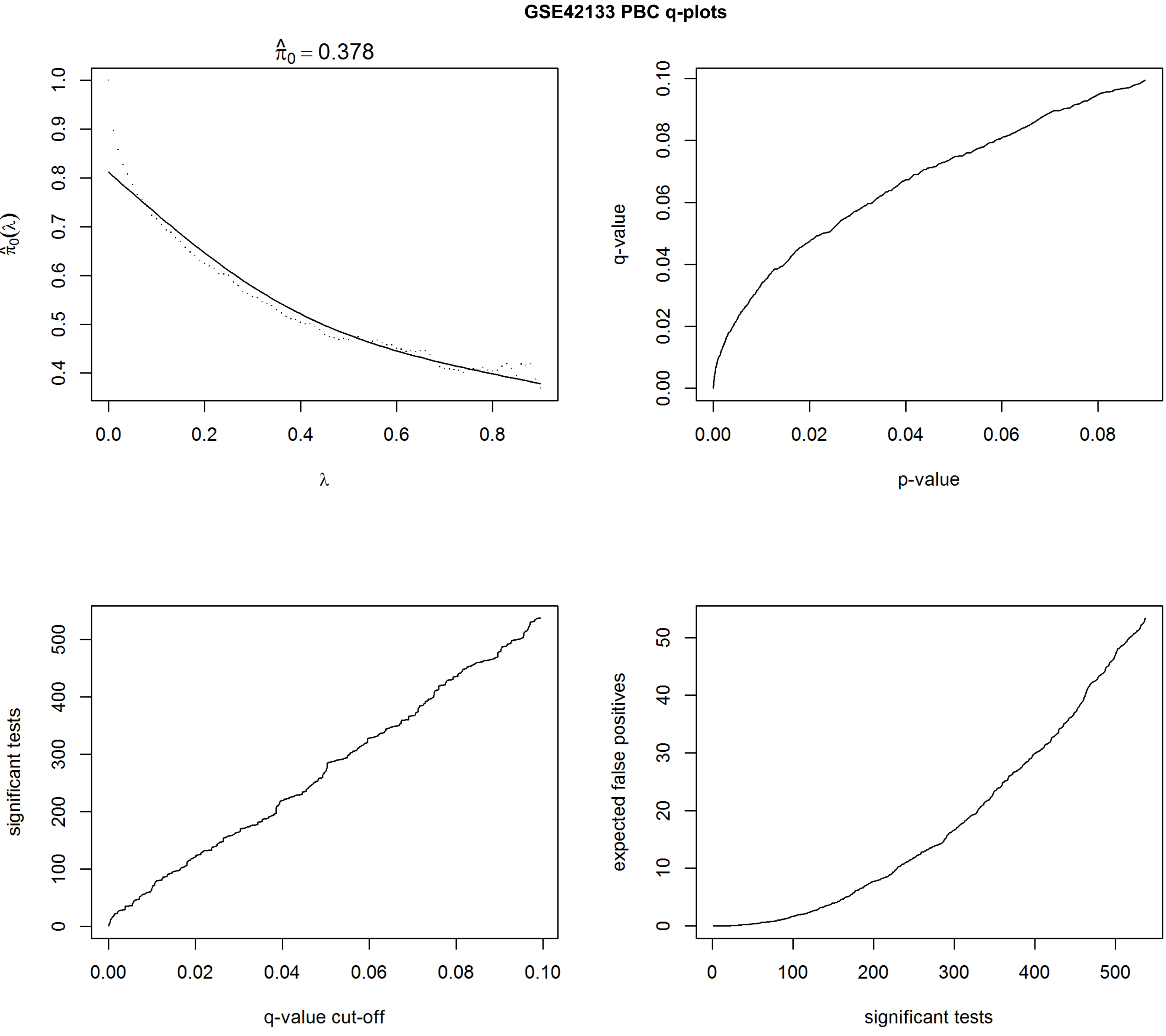

GSE42133 PBC q-plots
$\hat{\pi}_0 = 0.378$
$\hat{\pi}_0(\lambda)$
λ
1.0
0.9
0.8
0.7
0.6
0.5
0.4
0.0
0.2
0.4
0.6
0.8
q-value
p-value
0.10
0.08
0.06
0.04
0.02
0.00
0.00
0.02
0.04
0.06
0.08
significant tests
q-value cut-off
500
400
300
200
100
0
0.00
0.02
0.04
0.06
0.08
0.10
expected false positives
significant tests
50
40
30
20
10
0
0
100
200
300
400
500

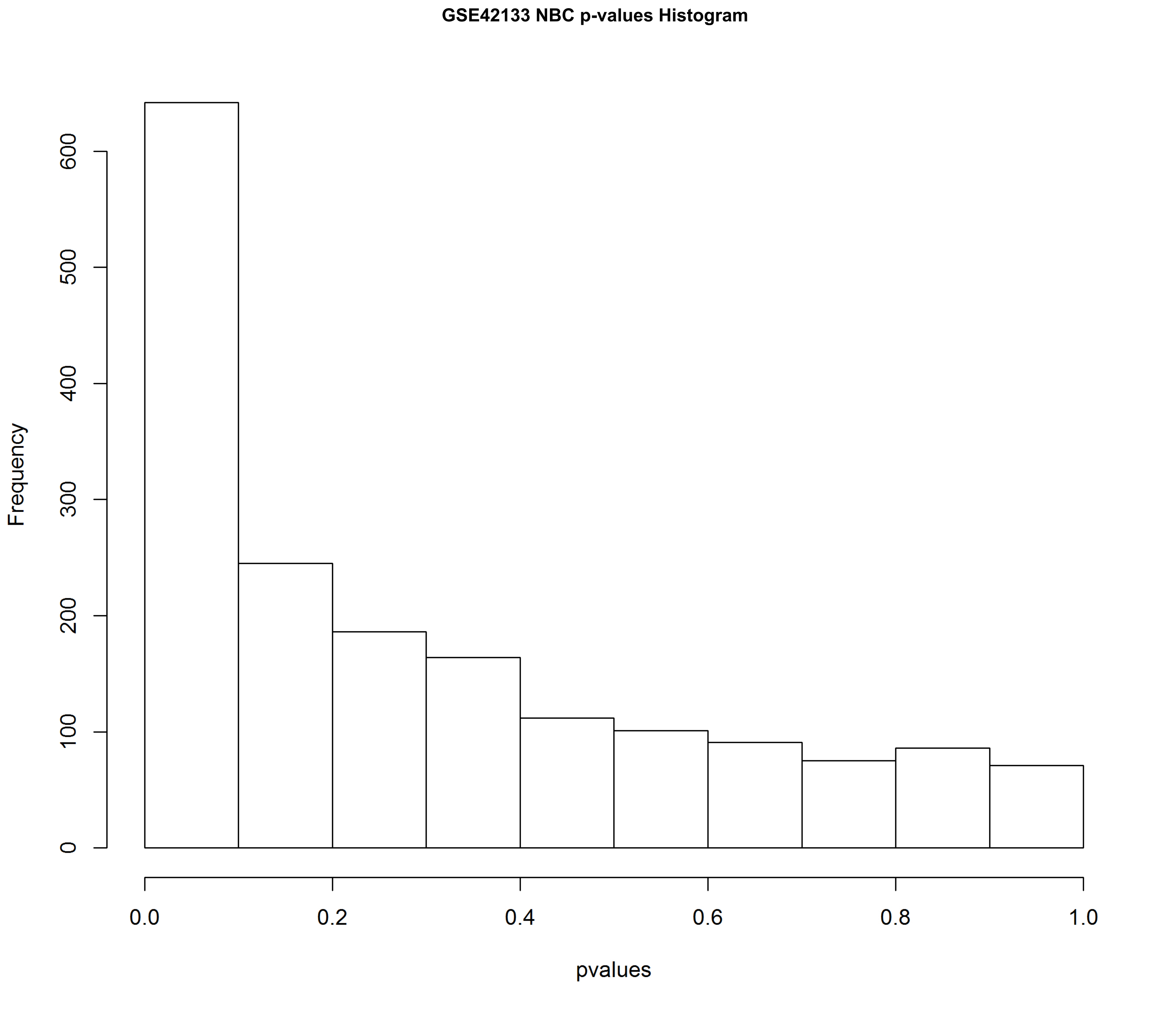
GSE42133 NBC p-values Histogram
Frequency
0
100
200
300
400
500
600
0.0
0.2
0.4
0.6
0.8
1.0
pvalues

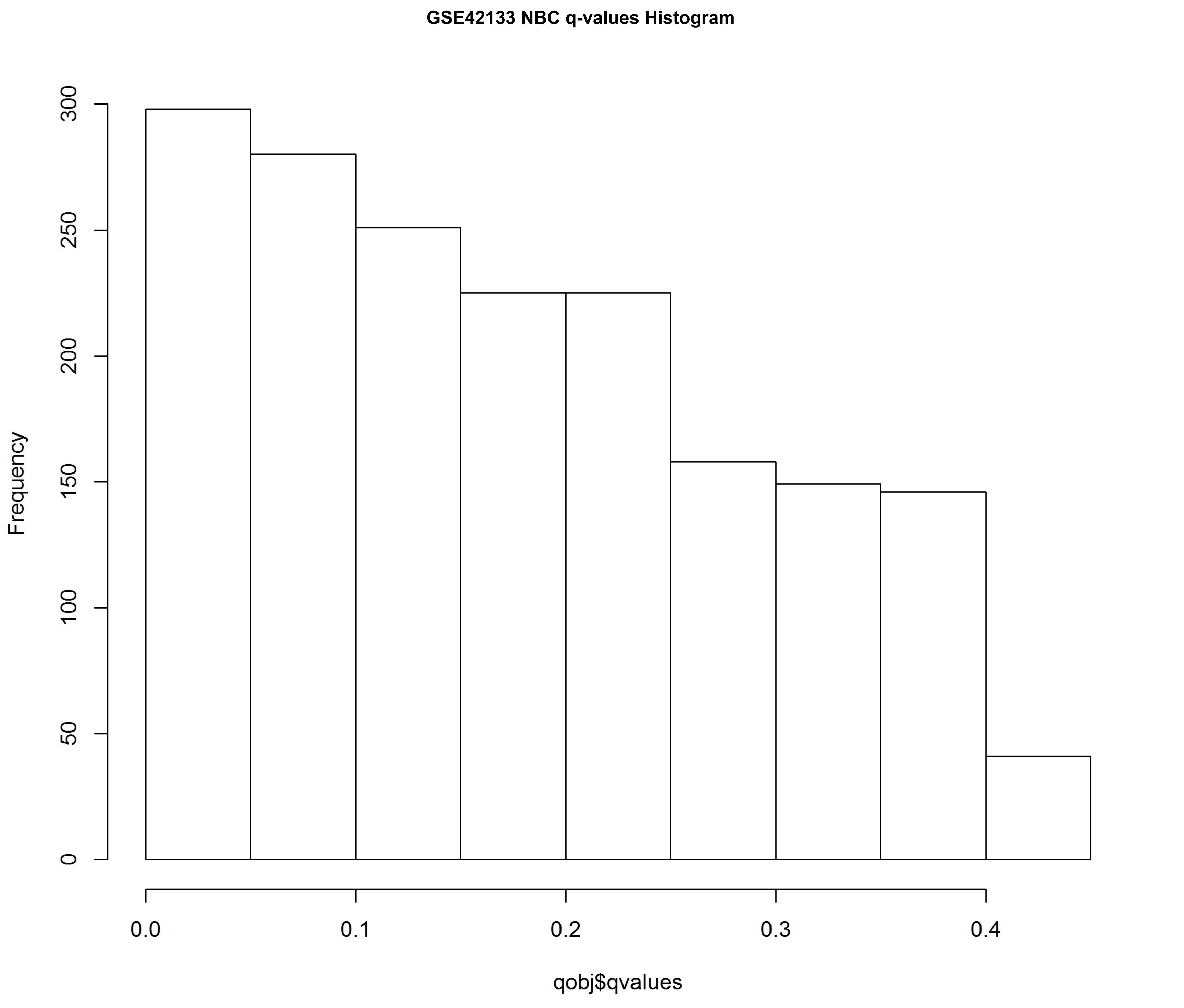
GSE42133 NBC q-values Histogram
Frequency
0
50
100
150
200
250
300
0.0
0.1
0.2
0.3
0.4
qobj$qvalues

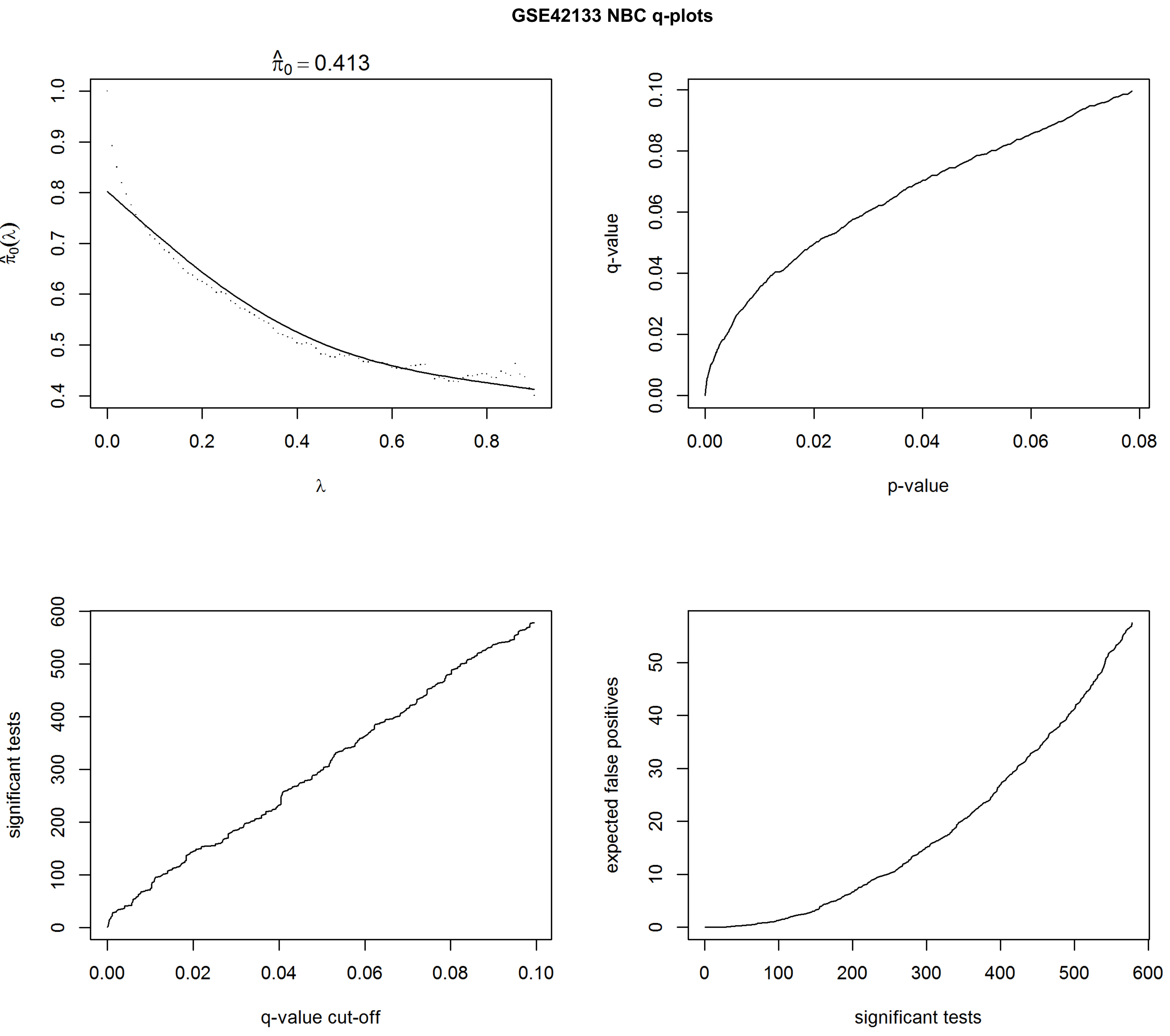
GSE42133 NBC q-plots
π̂0 = 0.413
π̂0(λ)
λ
0.0
0.2
0.4
0.6
0.8
0.4
0.5
0.6
0.7
0.8
0.9
1.0
q-value
p-value
0.00
0.02
0.04
0.06
0.08
0.10
significant tests
q-value cut-off
0
100
200
300
400
500
600
expected false positives
10
20
30
40
50
